# Macrophage‐Mediated Cellular Communication Networks in Lung Squamous Cell Carcinoma and Adenocarcinoma Revealed by Single‐Cell Sequencing

**DOI:** 10.1155/mi/9934067

**Published:** 2026-02-15

**Authors:** Xiaoyu Zhang, Yunlong Zhao, Yingying Wang, Xiaomin Yu, Hongyu Xia, Meiru Li, Xiu-An Yang

**Affiliations:** ^1^ Laboratory of Gene Engineering and Genomics, School of Basic Medical Sciences, Chengde Medical University, Chengde, 067000, China, cdmc.edu.cn; ^2^ Department of Biology Science, SDU-ANU Joint Science College, Shandong University (Weihai), Weihai, China, sdu.edu.cn; ^3^ Department of Emergency, Yantai Yuhuangding Hospital, Yantai, 264099, China, ytyhdyy.com; ^4^ Hebei Key Laboratory of Nerve Injury and Repair, Chengde Medical University, Chengde, 067000, China, cdmc.edu.cn

**Keywords:** lung adenocarcinoma, lung squamous cell carcinoma, macrophages, MIF signaling pathway, single-cell RNA sequencing

## Abstract

**Background:**

Lung cancer, particularly the non‐small cell lung cancer (NSCLC) subtypes lung squamous cell carcinoma (LUSC) and lung adenocarcinoma (LUAD), exhibits high heterogeneity and high mortality. This study aimed to explore their tumor microenvironment (TME) features, cellular interactions, and potential therapeutic targets.

**Methods:**

Using scRNA‐seq datasets (GSE200972, GSE117570, and GSE127465) and TCGA bulk RNA‐seq data, we performed cell clustering, pseudotime trajectory, cell–cell communication, and survival analyses. Batch correction and quality control were applied first, followed by cell type annotation with SingleR, copy number variation inference with InferCNV, and intercellular signaling investigation with CellChat.

**Results:**

Four epithelial signatures (S1–S4) with distinct gene expression profiles were identified, with S3 specific to LUSC and correlated with high malignancy. Pseudotime analysis revealed distinct differentiation trajectories: S2→S1→S3/S4 in LUSC and S4→S1→S2 in LUAD. In LUSC, S3 interacted with macrophages via the *SPP1* and *MIF* ligand–receptor pairs, involving the PI3K‐Akt pathways; in LUAD, S4 communicated with neutrophils through *MIF*, linked to interferon‐related pathways. Macrophages played a central role in the TME, with *SPP1-CD44* as a key ligand–receptor pair in LUSC and *RESISTIN-CAP1* in LUAD. Additionally, *CD44* and *CD74* expression correlated with prognosis in LUSC and LUAD, respectively.

**Conclusion:**

This study highlights subtype‐specific epithelial signatures, identifies key signaling pathways (e.g., *MIF*), and pinpoints candidate therapeutic targets (*CD44*, *CD74*). These discoveries shed new light on the distinct pathogenic mechanisms of LUSC and LUAD and provide actionable insights to facilitate the clinical translation of subtype‐specific personalized immunotherapies.

## 1. Introduction

Lung cancer is the most prevalent malignancy tumor worldwide and the leading cause of cancer‐associated deaths in males and the second most common cause of cancer death in females [1, 2]. Clinically, it is primarily classified into two main histological subtypes: non‐small cell lung cancer (NSCLC), which accounts for 80–85% of all lung cancer cases, and small cell lung cancer (SCLC), contributing to 10–15% [3, 4]. NSCLC is further subdivided into three main subtypes, namely lung squamous cell carcinoma (LUSC), lung adenocarcinoma (LUAD), and large cell carcinoma, with LUSC and LUAD comprising ~80% of NSCLC cases [4, 5].

Studies indicate that 91% of LUSC cases are associated with tobacco smoking [6, 7]. While smoking is the primary driver of lung cancer, 25% of patients—particularly nonsmoking women with LUAD—harbor distinct genetic alterations or are affected by other risk factors [8]. Epidermal growth factor receptor (*EGFR*) mutations occur in ~10% of lung cancer cases, with higher incidence in Asian populations and nonsmoking women [9]. In addition to *EGFR* mutations, proto‐oncogene tyrosine‐protein kinase *ROS1* rearrangements and mesenchymal‐epithelial transition (*MET*) oncogene alterations are key oncogenic events; unlike *EGFR* mutations, these alterations render tumors responsive to the tyrosine kinase inhibitor (TKI) crizotinib [9]. These rare alterations are LUAD‐specific, thus supporting the recommendation of routine testing for *EGFR* mutations and *ROS1* rearrangements in all advanced LUAD patients [10].

Stage I NSCLC patients have a 5‐year overall survival rate of ~80%, versus 13–60% for stage II–III patients [11, 12]. Radiotherapy and chemotherapy have moderately improved survival, but their side effects severely compromise patient health—specifically inducing normal cell cytotoxicity, with grade 3/4 toxicities occurring in 66% of cases (32% grade 4) and neutropenia as the most common adverse event [13]. Moreover, chemotherapy also increases venous thromboembolism risk in NSCLC patients [14].

In recent years, NSCLC treatment has evolved from empirical cytotoxic chemotherapy to subtype‐specific precision‐targeted therapeutic regimens tailored to LUSC and LUAD molecular subtypes [15]. Historically, these two subtypes were treated similarly [13], but they exhibit distinct mutation spectra and somatic copy number alterations: EGFR and KRAS mutations, common in LUAD, are rare in LUSC [16, 17]. Targeted therapies based on novel cancer biomarkers have significantly improved progression‐free survival and overall survival in LUAD patients [18], yet early trials show poor efficacy in LUSC [19–21]. Thus, identifying subtype‐specific therapeutic targets is critical for developing effective treatments for both subtypes.

Single‐cell RNA sequencing (scRNA‐seq) quantifies molecular changes at single‐cell resolution, clarifying individual cell responses to tissue alterations and providing a robust framework for studying disease pathophysiology [22, 23]. As a groundbreaking tool, it deciphers intercellular interactions and advances clinical disease management [24, 25]. Specifically, in oncology, it reveals cell–cell crosstalk, identifies malignant cell types, and enhances understanding of tumor initiation and progression [26]. In this study, we obtained LUSC and LUAD scRNA‐seq datasets from public databases and performed analyses—including cell–cell communication profiling—to identify key cell types involved in their development and progression.

## 2. Materials and Methods

### 2.1. scRNA‐Seq Data Processing

The scRNA‐seq dataset GSE200972 was obtained from the Gene Expression Omnibus (GEO, https://www.ncbi.nlm.nih.gov/gds/?term=) database. This dataset includes a total of 11 pathological tissue samples of LUSC and LUAD derived from four patients. Specifically, two distinct tumor lesions were sampled from the lungs of each LUSC patient and the first LUAD patient. For the second LUAD patient, two separate tissue samples were collected from two individual tumor foci; the third LUAD patient provided three tumor tissue samples. Datasets GSE117570 and GSE127465 were utilized as validation cohorts, comprising 7 LUSC and 15 LUAD cases. Detailed clinical and pathological characteristics of the patients are in Supporting Information 1: Table S1. Prior to downstream analyses, a standardized computational pipeline was established using Seurat (v4.3.0). To ensure analytical rigor, stringent quality control (QC) criteria were implemented as follows: cells expressing fewer than 100 or more than 50,000 genes were excluded. High‐quality cells were defined by three thresholds: unique molecular identifier (UMI) counts > 500, mitochondrial UMI ratio < 10%, and red blood cell gene ratio < 1%. Technical doublets were computationally detected with DoubletFinder and subsequently removed. Gene expression profiles were normalized and scaled using Seurat’s built‐in functions: NormalizeData (log‐normalization with a scale factor of 10,000) and ScaleData (linear scaling with regression of mitochondrial gene percentage). The top 3000 highly variable genes (HVGs) were identified via the FindVariableFeatures function and used as input for principal component analysis (PCA). PCA was performed using the RunPCA function, followed by batch effect correction with the RunHarmony function from the Harmony package. Cellular clustering was conducted using the FindNeighbors and FindClusters functions with the top 40 principal components (PCs), and dimensionality reduction was achieved via the Uniform Manifold Approximation and Projection (UMAP) method. Subsequently, cell type annotation was performed using the “SingleR” algorithm. To verify the reliability of the annotated cell subtypes, the marker genes of each cluster were compared with previously reported signatures in the literature. This cross‐validation confirmed the accuracy of the cell classification results [27].

### 2.2. Gene Set Functional Analysis

The gene sets MSigDB_Hallmark_2020 and WikiPathway_2021_Human were retrieved from the Enrichr database using the R package “Enrichr.” Subsequently, gene set enrichment analysis of marker genes was performed using the R packages “Enrichr” and “clusterProfiler.” To quantify the activity levels of the enriched pathways across distinct cell clusters, the *AddModuleScore* function embedded in the R package *Seurat* was utilized to calculate pathway enrichment scores for each gene set. The resultant pathway activity profiles were visualized as heatmaps.

### 2.3. InferCNV Analysis

The copy number variations (CNVs) in epithelial cells were analyzed in R using the “InferCNV” package. The analysis was performed strictly following the official workflow to generate the raw read count matrix, cell type annotation file, and gene‐chromosome position annotation file. T cells were designated as the reference normal cells, with the denoising threshold set to 2.5; all other parameters of the InferCNV package remained at their default settings.

### 2.4. Cell–Cell Communication Analysis

Cell–cell communication was analyzed in R using the CellChat package, with a random subset of 20,000 cells selected for the analysis. Detailed scrutiny of the secreted signaling category in CellChatDB uncovered overexpressed genes and ligand–receptor pairs indicative of potential intercellular communication networks. To infer these networks, cell groups within specific populations containing fewer than 10 cells were excluded. After exclusion of these small cell groups, visual representations, including interaction counts, strengths, and diagrams of individual signaling pathways, were generated. Cell types were categorized by evaluating inbound and outbound signals using the “NMF” package, which identified ligand–receptor gene pairs closely associated with each cell type based on these signaling pathways.

### 2.5. Pseudotime Trajectory Analysis

Pseudotime analysis was performed using Monocle (version 2.26.0) to infer the developmental and evolutionary trajectories of cell subtypes. Monocle prioritized genes exhibiting higher expression variability across cells for feature selection in trajectory inference, and the top 1000 HVGs were selected for this analysis. As a standard preprocessing step in scRNA‐seq analysis, HVG selection reduces data dimensionality, mitigates noise, and emphasizes genes with the most prominent expression changes across cells—changes that are often associated with cell differentiation or state transitions. Following this, the DDRTree algorithm in the reduceDimension() function was applied to construct a minimum spanning tree, which connects cells while minimizing the total edge weights and facilitates the delineation of cell differentiation trajectories. Subsequently, the inferred trajectory was visualized using the plot_cell_trajectory() function.

### 2.6. Bulk Dataset Selection and Preparation

RNA‐seq data (raw read counts) for The Cancer Genome Atlas Lung Squamous Cell Carcinoma (TCGA‐LUSC) and The Cancer Genome Atlas Lung Adenocarcinoma (TCGA‐LUAD) were obtained from The Cancer Genome Atlas Program (TCGA). Following data acquisition, differential analysis was independently conducted for each tumor type using the criteria: fold change > 1 and *p*‐value < 0.05. Subsequently, pathways derived from CellChat were utilized to perform correlation analysis of ligand–receptor pairs identified from the differential analysis. Finally, survival analysis was performed on these ligand–receptor pairs for each tumor type, with samples dichotomized into high and low expression groups based on median expression to identify potential therapeutic targets.

## 3. Results

### 3.1. scRNA‐Seq Reveals Diverse Cell Types and Tumor Heterogeneity in LUSC and LUAD

To investigate the tumor heterogeneity of LUSC and LUAD, GEO data (GSE200972) were downloaded for subsequent scRNA‐seq analysis. After rigorous batch effect correction and QC, a total of 66,197 cells were retained and subjected to cell type annotation using the SingleR algorithm. As illustrated in Figure 1A,B, these cells were classified into nine distinct lineages, with each cell type defined by its canonical marker genes: natural killer (NK) cells (*IL18R1* and *KLRG1*), epithelial cells (*KRT18* and *SFTPD*), T cells (*CD3D* and *CD3E*), B cells (*CD79A* and *MS4A1*), macrophages (*CD68* and *CD163*), fibroblasts (*DCN* and *LUM*), dendritic cells (DCs) (*CD40* and *CD80*), endothelial cells (*RAMP2* and *VWF*), and neutrophils (*CSF3R* and *FCGR3B*) (Supporting Information 2: Table S2).

Figure 1scRNA‐seq reveals diverse cell types and tumor heterogeneity in LUSC and LUAD. (A) UMAP plots showing the clustering of 66,197 cells from LUSC and LUAD samples into nine distinct cell types: NK cells, epithelial cells, T cells, B cells, macrophages, fibroblast cells, DCs, endothelial cells, and neutrophils. Cells were annotated based on marker genes using the ‘SingleR’ function. (B) Violin plots depicting the expression levels of marker genes for each cell type. (C) Bar plot illustrating the relative abundance of each cell type in LUSC and LUAD samples.

**Figure:**
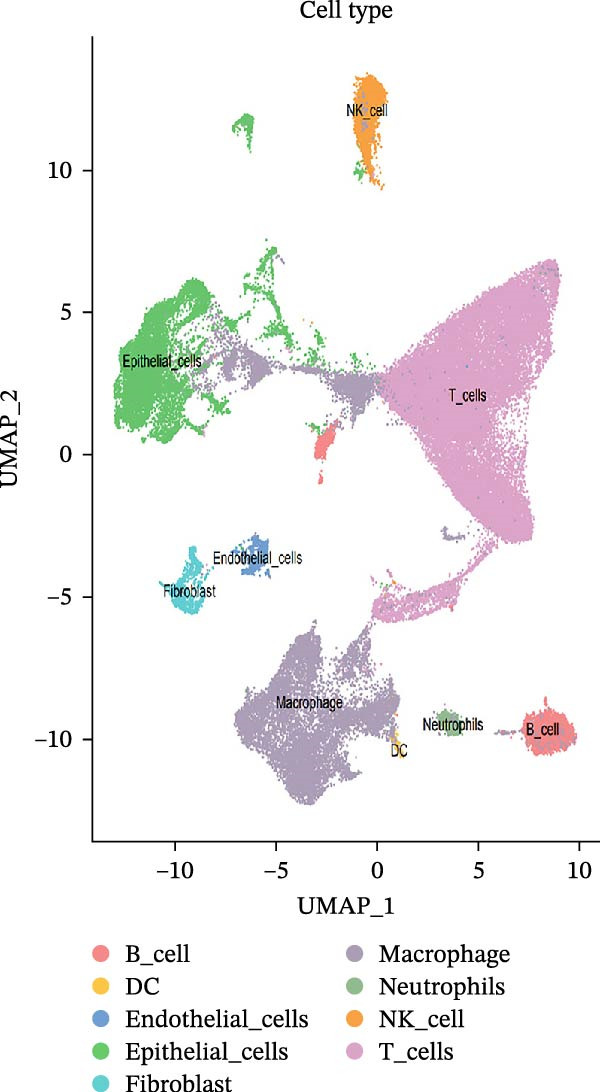
(A)

**Figure:**
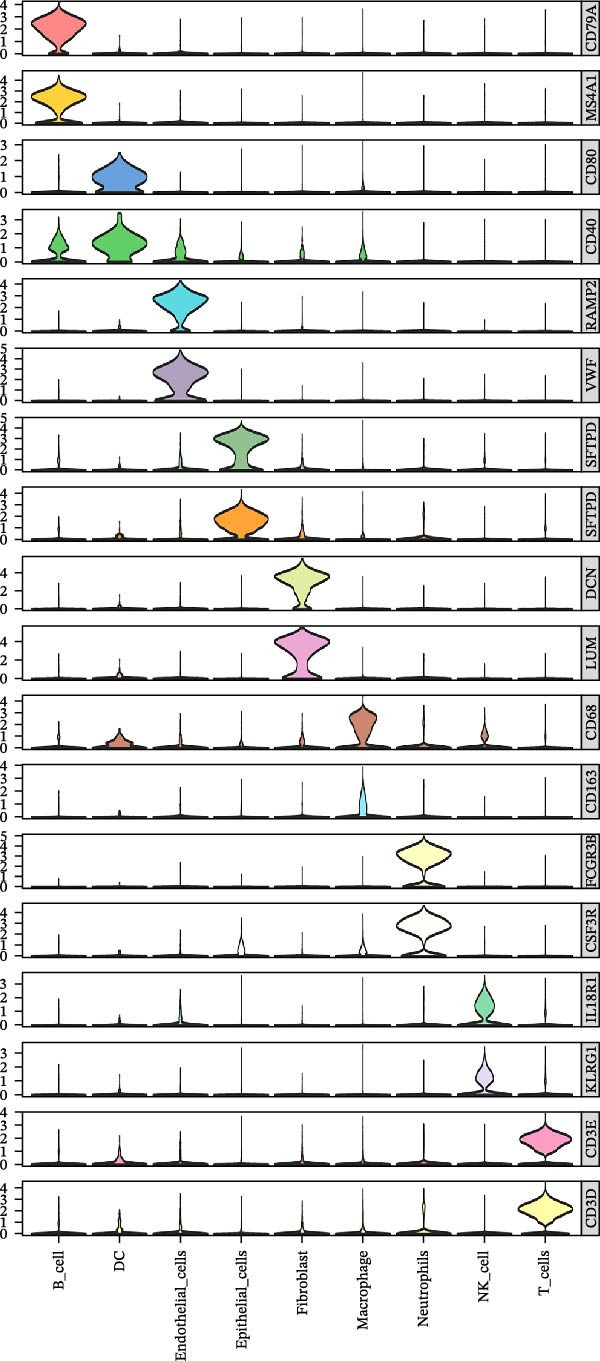
(B)

**Figure:**
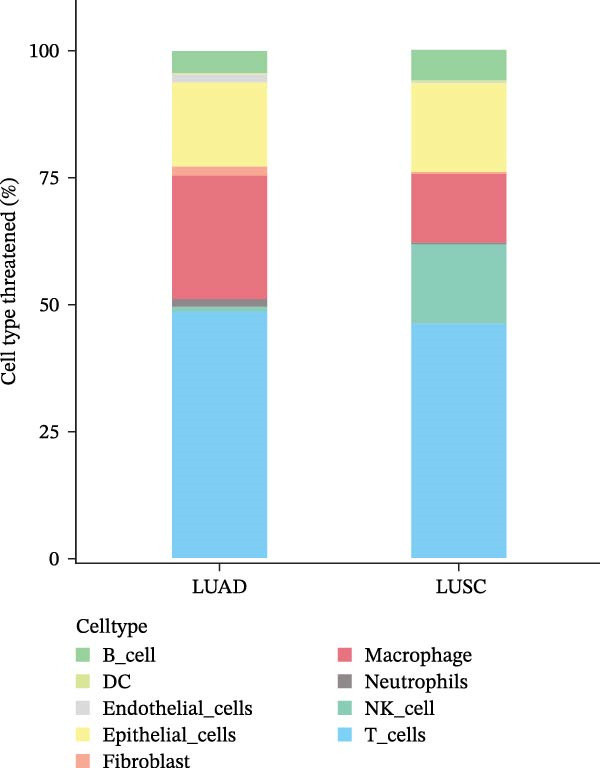
(C)

The cellular composition bar chart clearly delineated the intertumoral composition differences between LUSC and LUAD (Figure 1C). While both tumor types shared comparable proportions of epithelial cells, a notable discrepancy was observed in endothelial cells—a cell population was detectable in LUAD yet completely absent in LUSC, a finding that may underpin the divergent pathological and biological characteristics of the two cancer subtypes. Notably, consistent with prior literature, LUSC is primarily derived from bronchial epithelial cells [28], whereas LUAD predominantly originates from bronchial mucosal epithelium, with a minor subset arising from bronchial glandular epithelium, thus representing a form of peripheral lung cancer with unique histopathological traits [29]. Our analysis further revealed abundant infiltration of immune cells in both LUSC and LUAD, albeit with subtype‐specific variations in immune cell composition and abundance. Specifically, macrophages and T cells exhibited higher prevalence in LUAD, whereas LUSC was characterized by elevated levels of NK cells, macrophages, and T cells.

### 3.2. Distinct Epithelial Cell Signatures Correlate With Tumor malignancy in LUSC and LUAD

It is reported that LUSC predominantly arises from the malignant transformation of bronchial epithelial cells [28, 30]. To investigate the roles of epithelial cells in both LUSC and LUAD, epithelial cells were isolated for in‐depth characterization. Following batch correction and dimensionality reduction, 10,759 epithelial cells were identified and clustered into 18 distinct groups (Supporting Information 3: Figure S1A and Supporting Information 4: Table S3). Subsequently, the “NMF” algorithm was employed to classify these epithelial cells into four molecular signatures (Figure 2A–C and Supporting Information 3: Figure S1B). Our results revealed that Signature 3 (S3) was exclusive to LUSC, whereas the other signatures were shared between LUSC and LUAD, albeit with marked differences in their proportional distributions; notably, LUSC exhibited a significantly lower abundance of S1 cells (Supporting Information 3: Figure S1B). Within LUSC, the genes *CES1* and *AKR1C2* were specifically upregulated in the S3 subpopulation. This lineage‐specific association was further validated in the GSE117570 and GSE127465 datasets, which confirmed the predominant enrichment of the S3 subpopulation in LUSC (Supporting Information 5: Figure S2A–D).

Figure 2Distinct epithelial cell signatures correlate with tumor malignancy in LUSC and LUAD. (A,B) The epithelial cells were classified into four signatures by “NMF” algorithm. (C) UMAP plots showing the four distinct signatures of 10,759 epithelial cells of LUSC and LUAD. (D) Heatmap showing the variability of different epithelial cell signatures as determined by InferCNV analysis, with T cells used as reference cells. The S3 characteristic in epithelial cells exhibited the highest degree of variability across various types of epithelial cells. (E) Heatmap showing the pathway enrichment analysis of the four epithelial cell signatures using the ‘AddModuleScore’ function from the ‘Seurat’ package.

**Figure:**
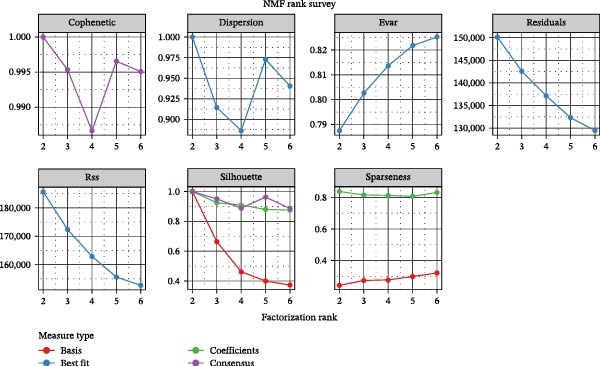
(A)

**Figure:**
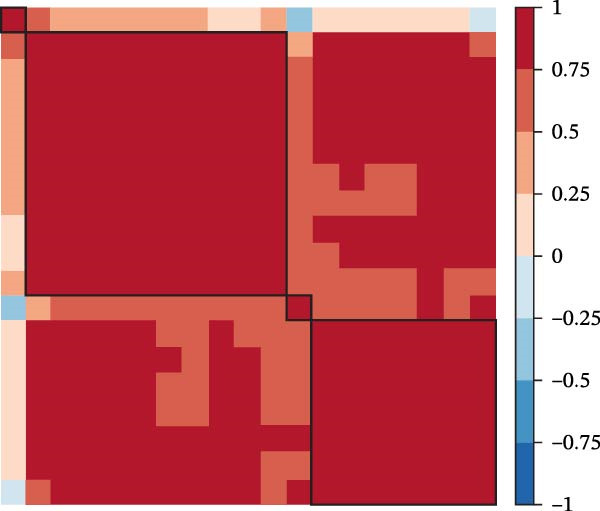
(B)

**Figure:**
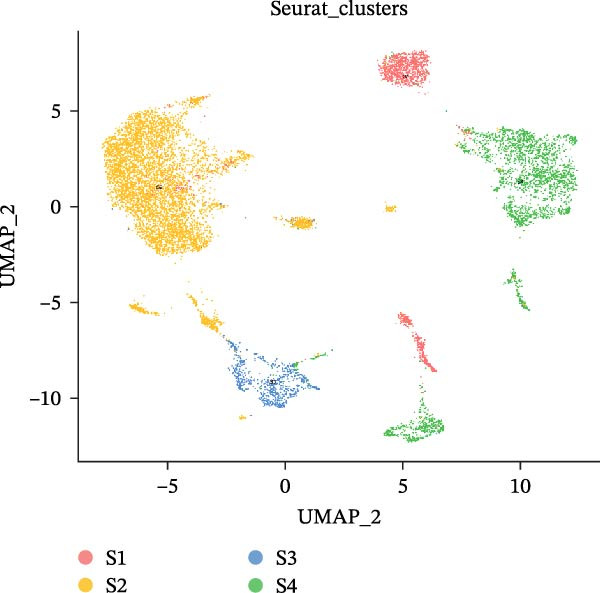
(C)

**Figure:**
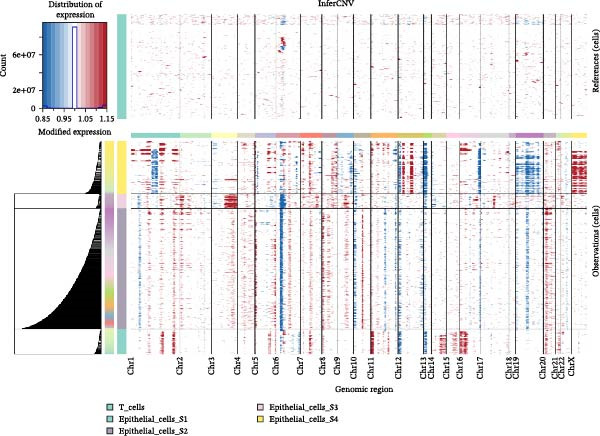
(D)

**Figure:**
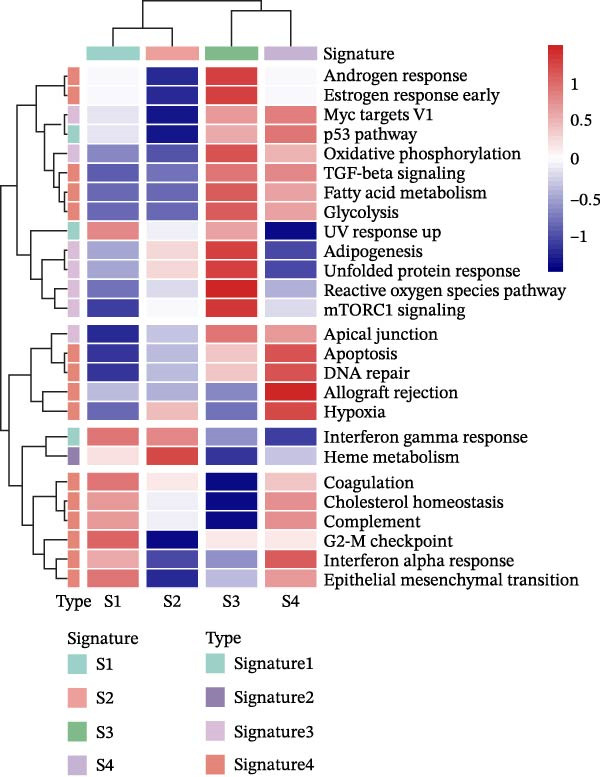
(E)

Cell composition profiling across both tumor types demonstrated that T cells constituted the most abundant immune cell subset. Furthermore, T cells exhibited significantly fewer genomic CNVs during tumor initiation and progression compared with epithelial cells. Accordingly, T cells were designated as the reference cell type in the “InferCNV” analysis to assess CNV heterogeneity across epithelial cell signatures and its correlation with tumor malignancy [31]. Genome‐wide CNV profiling of all epithelial cells revealed that the S3 subpopulation exhibited the highest CNV variability (Figure 2D and Supporting Information 3: Figure S1C), indicative of heightened malignancy. Taken together, these findings identified S3 as an LUSC‐specific epithelial cell subpopulation. Consistent with this observation, the S3 subpopulation also showed elevated CNV variability in the GSE117570 and GSE127465 datasets (Supporting Information 5: Figure S2E,F). These findings suggested that epithelial cell‐intrinsic genetic alterations were likely a primary driver of LUSC oncogenesis, a conclusion that corroborates prior research [30].

To elucidate the pathway activity landscapes associated with these four epithelial cell signatures, we utilized the AddModuleScore function implemented in the Seurat package, with the results visualized as heatmaps (Figure 2E). Pathway enrichment analyses revealed that S1 was significantly involved in UV response up and interferon gamma response pathways; S2 was linked to heme metabolism; S3 was enriched for pathways governing reactive oxygen species, mTORC1 signaling, and the unfolded protein response; and S4 was associated with the allograft rejection, DNA repair, and hypoxia pathways. Thus, this study identified an LUSC‐specific epithelial cell subpopulation (S3) characterized by high malignancy and distinct pathway activation profiles and provided evidence supporting the pivotal role of epithelial cell genetic alterations in LUSC tumorigenesis.

### 3.3. Pseudo‐Temporal Transition Trajectory of Epithelial Tumor Cells in LUSC

To explore the differentiation evolutionary trajectories of epithelial cells in LUSC and the influence of the top 1000 HVGs on tumor development, pseudotime trajectory analysis of the four signatures was conducted using the “monocle2” package in R. The analysis revealed that epithelial cells could be categorized into approximately three differentiation stages (Figure 3A,B). S1 cells exhibited a distinct distribution pattern across differentiation, whereas the other three clusters spanned all stages (Figure 3C). Although S1 appeared to differentiate earlier, the sequential order of S2, S3, and S4 remained unclear. To clarify this, we used the “monocle2” package to investigate expression changes in HVGs during cell differentiation. The pseudotime ridge plot in Figure 3D indicated that S2 occupied the initial differentiation stage, S1 the middle phase, and S3 and S4 the terminal stage. The heatmap in Figure 3E illustrates the expression patterns of HVGs, consistent with the results in Figure 3D. At the individual gene level, *CXCL1*, *CXCL3*, *LTF*, and *AGER* were upregulated in the early stage (Figure 3F). Additionally, *SAA1*, *SAA2*, *CCL5*, and *CXCL6* showed increased expression in the mid‐stage (Figure 3G), while *BIRC5*, *TCHH*, *IGFBP3*, and *KRT14* were upregulated in the late differentiation phase (Figure 3H).

Figure 3Pseudo‐temporal trajectory analysis of epithelial cells in LUSC. (A,B) Pseudo‐temporal trajectory of the four epithelial cell signatures in LUSC, with the trajectory colored according to the pseudo‐time. (C) Distribution of the four epithelial cell signatures across the pseudo‐time trajectory. (D) Ridge plot showing the expression patterns of highly variable genes across the pseudo‐time trajectory. (E) Heatmap showing the expression patterns of selected highly variable genes in the four epithelial cell signatures across the pseudo‐time trajectory. (F) The expression patterns of selected highly variable genes (CXCL1, CXCL3, LTF, and AGER) in epithelial cell S2 of LUSC across the pseudo‐time trajectory. (G) The expression patterns of selected highly variable genes (SAA1, SAA2, CCL5, and CXCL6) in epithelial cell S1 of LUSC across the pseudo‐time trajectory. (H) The expression patterns of selected highly variable genes (BIRC5, TCHH, IGFBP3, and KRT14) in epithelial cells S3 and S4 of LUSC across the pseudo‐time trajectory.

**Figure:**
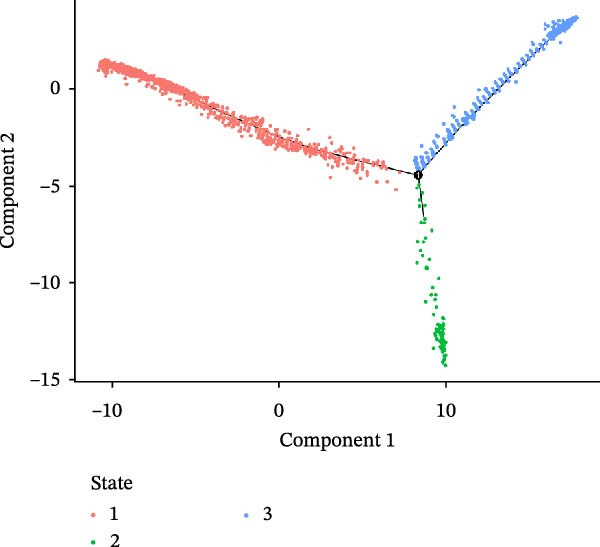
(A)

**Figure:**
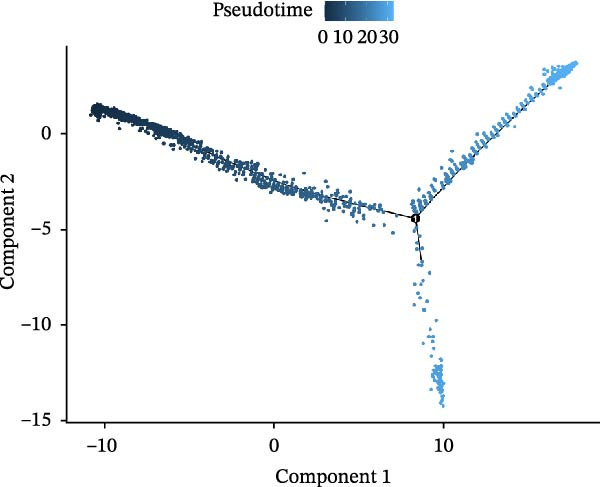
(B)

**Figure:**
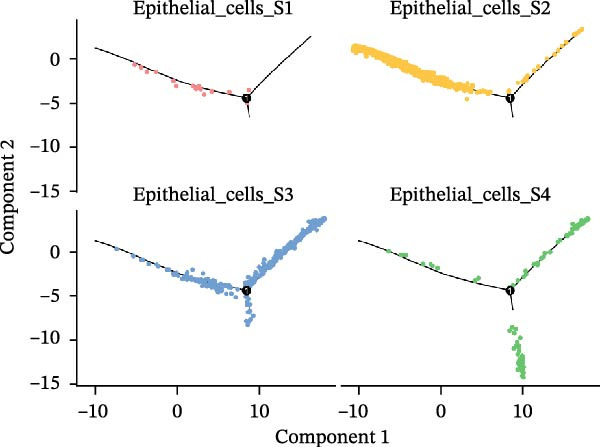
(C)

**Figure:**
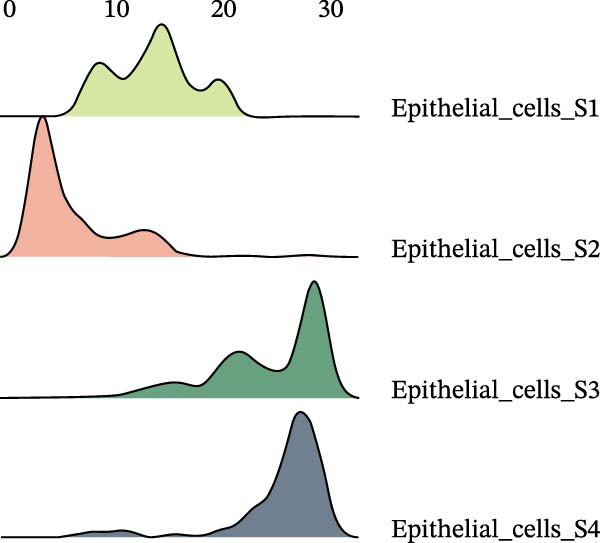
(D)

**Figure:**
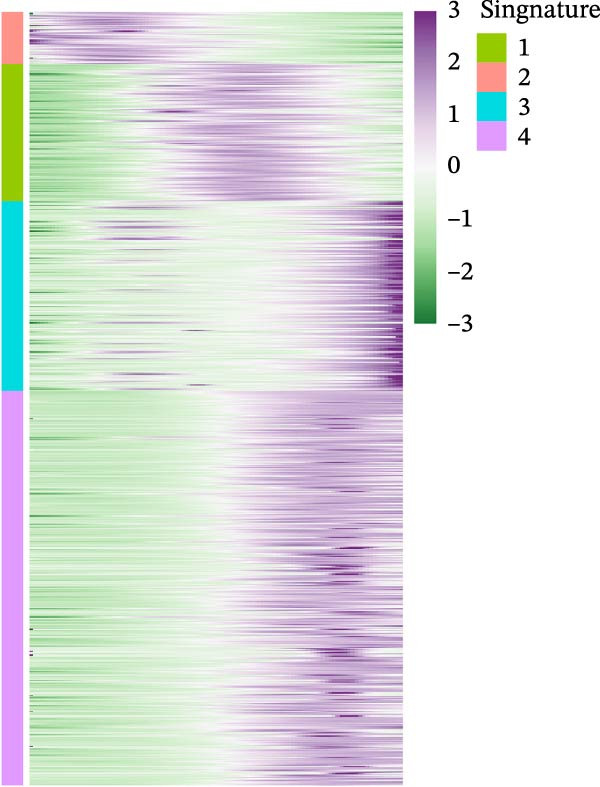
(E)

**Figure:**
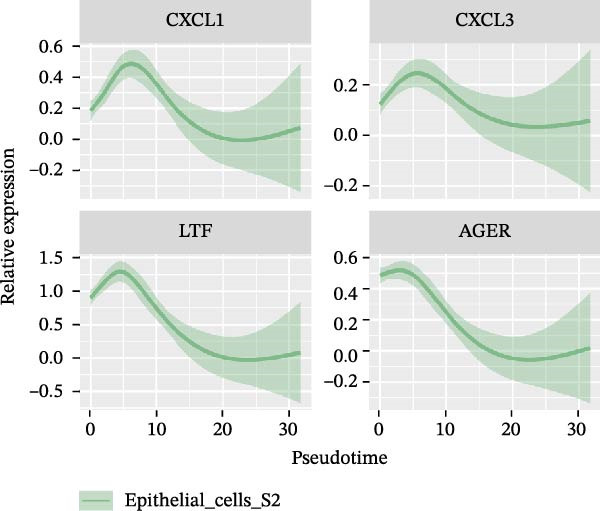
(F)

**Figure:**
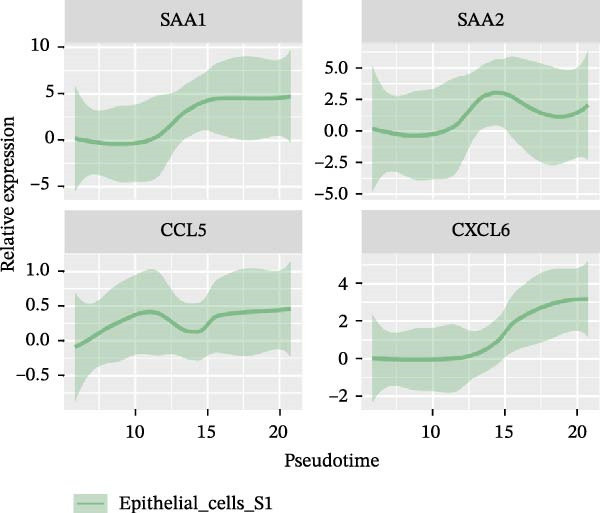
(G)

**Figure:**
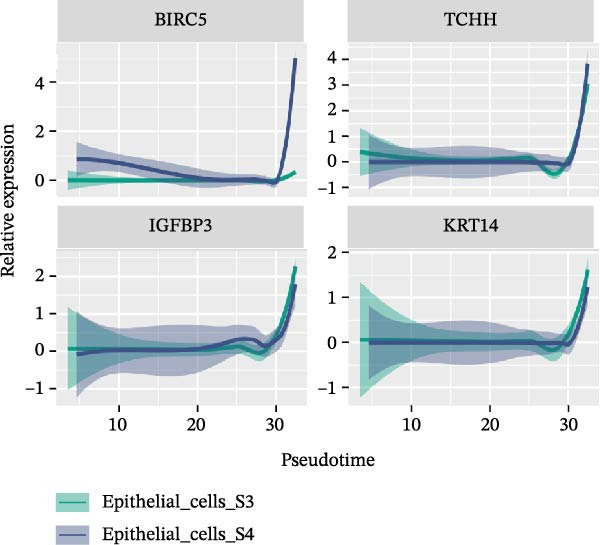
(H)

Pathway enrichment analysis showed that the early‐stage upregulated genes (*CXCL1*, *CXCL3*, *LTF*, and *AGER*) were enriched in pathways including senescence and autophagy in cancer, NRF2 Pathway, and Cytokines and Inflammatory Response (Supporting Information 6: Figure S3A). These pathways are activated under initial carcinogenic stress and regulate cell fate decisions (death, senescence, or mutation accumulation). The mid‐stage upregulated genes (*SAA1*, *SAA2*, *CCL5*, and *CXCL6*) were enriched in TNF‐α signaling via NF‐κB, angiogenesis, epithelial mesenchymal transition, and chemokine signaling pathway (Supporting Information 6: Figure S3B), which directly drive tumor expansion and survival as core mediators of this stage. The late‐stage upregulated genes (*BIRC5*, *TCHH*, *IGFBP3*, and *KRT14*) were enriched in pathways such as epithelial mesenchymal transition, hypoxia, the RAC1/PAK1/p38/MMP2 pathway, photodynamic therapy‐Induced NF‐κB survival signaling, and apoptosis modulation and signaling pathways (Supporting Information 6: Figure S3C). These pathways promote late‐stage tumor aggressiveness, which is a major contributor to treatment failure and patient death. Collectively, pseudotime analysis stratified the four signatures into approximately three differentiation stages and identified key genes that may modulate LUSC progression.

### 3.4. Pseudo‐Temporal Transition Trajectory of Epithelial Tumor Cells in LUAD

To gain a deeper insight into the key genes driving LUAD, we adopted the same analysis strategy as for LUSC. In LUAD, epithelial cells were stratified into three distinct states (Figure 4A,B). Epithelial cells expressing the three signatures were detected across all states; however, the differentiation sequence of cells corresponding to each signature remained undefined (Figure 4C). To elucidate the evolutionary order of these signature cells, we applied the LUSC‐matched approach to analyze HVGs in LUAD. The pseudotime ridge plot (Figure 4D) and heatmap (Figure 4E) indicated that S4 corresponded to the early differentiation stage, S1 to the intermediate phase, and S2 to the terminal stage. Analysis of the top 50 HVGs revealed that *CXCL14*, *DST*, *IDO1*, and *GPX2* were upregulated in the early stage (S4, Figure 4F); *SPP1*, *KRT17*, *IGFBP3*, and *CXCL8* in the mid‐stage (S1, Figure 4G); and *TOP2A*, *GCLC*, *SPINK1*, and *CAPS* in the terminal differentiation phase (S2, Figure 4H).

Figure 4Pseudo‐temporal trajectory analysis of epithelial cells in LUAD. (A,B) Pseudo‐temporal trajectory of the four epithelial cell signatures in LUAD, with the trajectory colored according to the pseudo‐time. (C) Distribution of the three epithelial cell signatures across the pseudo‐time trajectory. (D) Ridge plot showing the expression patterns of highly variable genes across the pseudo‐time trajectory. (E) Heatmap showing the expression patterns of selected highly variable genes in the three epithelial cell signatures across the pseudo‐time trajectory. (F) The expression patterns of selected highly variable genes (CXCL14, DST, IDO1, and GPX2) in epithelial cell S2 of LUAD across the pseudo‐time trajectory. (G) The expression patterns of selected highly variable genes (SPP1, KRT17, IGFBP3, and CXCL8) in epithelial cell S1 of LUAD across the pseudo‐time trajectory. (H) The expression patterns of selected highly variable genes (TOP2A, GCLC, SPINK1, and CAPS) in epithelial cells S3 and S4 of LUAD across the pseudo‐time trajectory.

**Figure:**
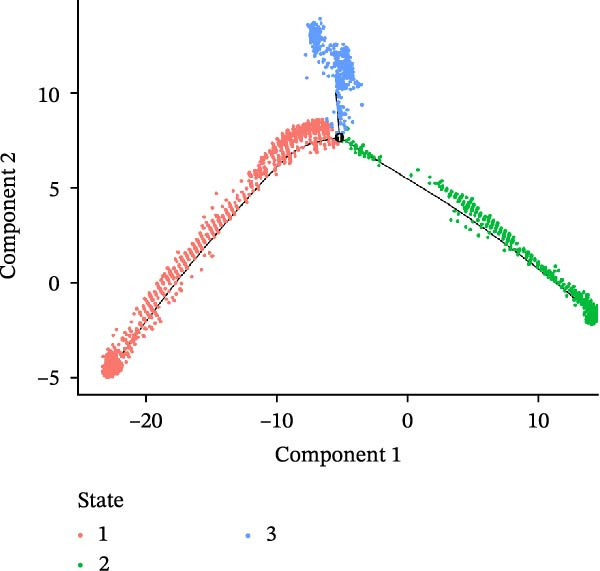
(A)

**Figure:**
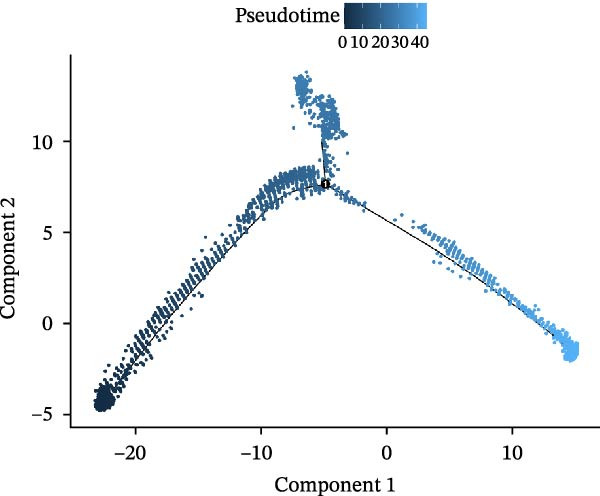
(B)

**Figure:**
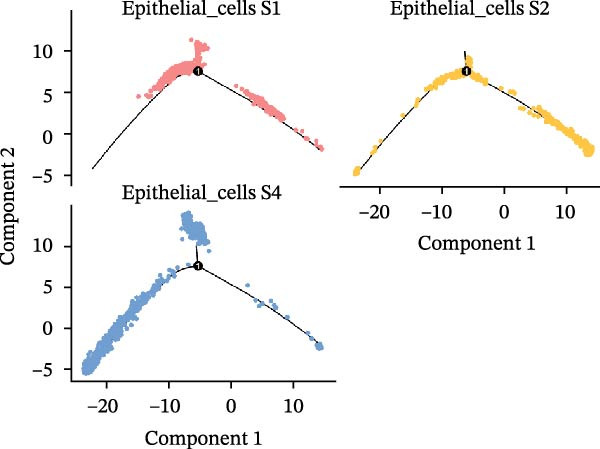
(C)

**Figure:**
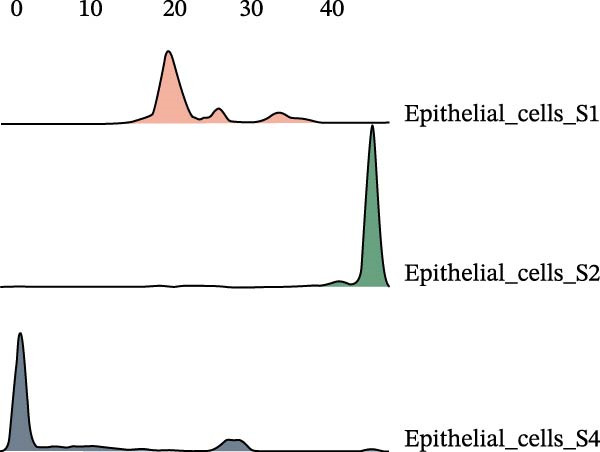
(D)

**Figure:**
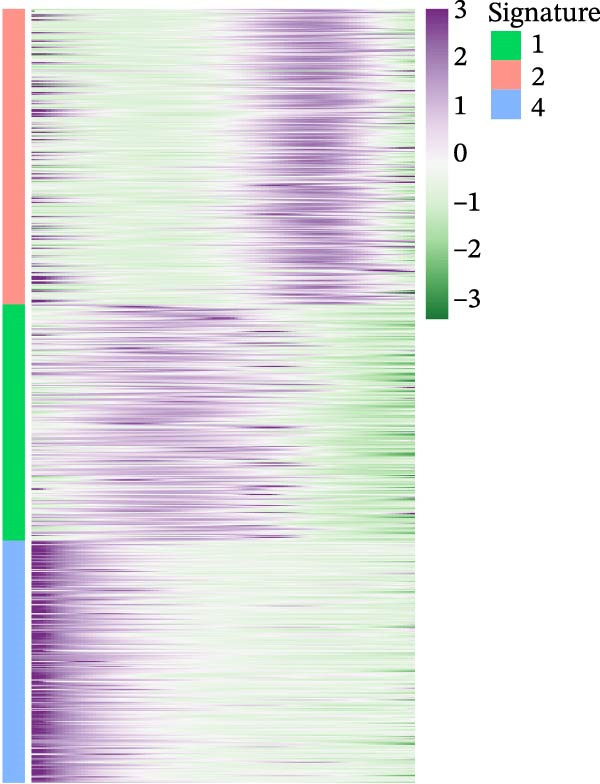
(E)

**Figure:**
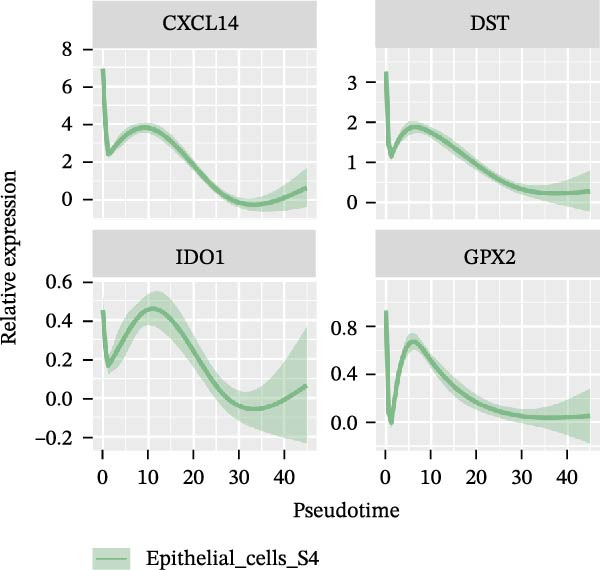
(F)

**Figure:**
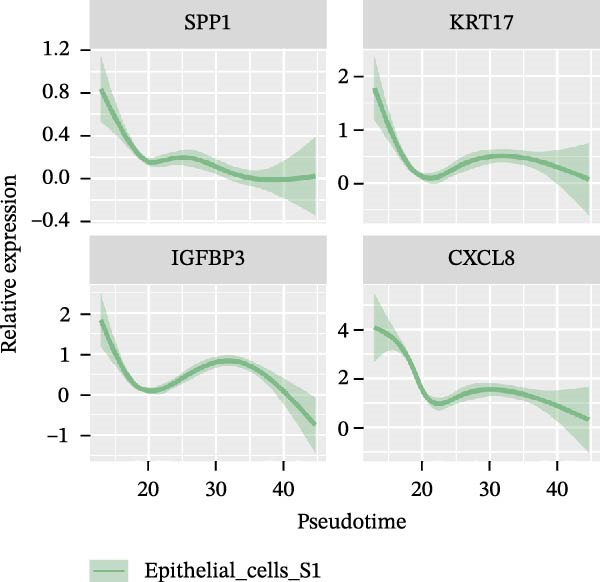
(G)

**Figure:**
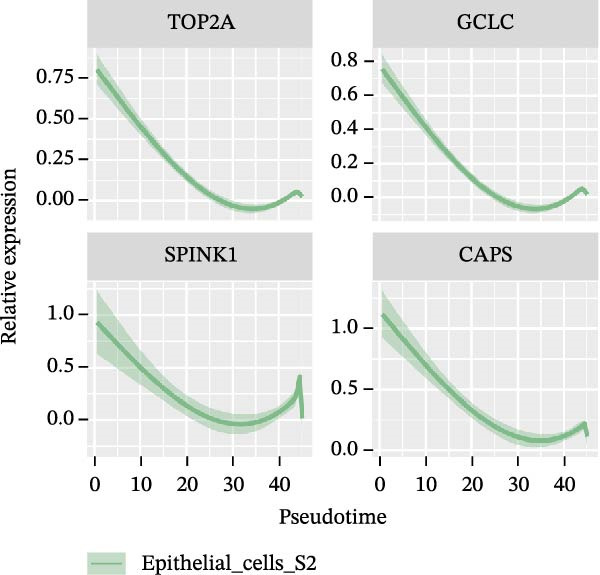
(H)

Pathway enrichment analysis showed that the early‐stage upregulated genes (*CXCL14*, *DST*, *IDO1*, and *GPX2*) were enriched in the p53 pathway, senescence and autophagy in cancer, the NRF2 pathway, glutathione metabolism, and the kynurenine pathway, linking to cellular senescence (Supporting Information 6: Figure S3D). These pathways are activated under initial carcinogenic stress (e.g., carcinogens, oxidative stress) and act as the first line of defense against tumorigenesis. The mid‐stage upregulated genes (*SPP1*, *KRT17*, *IGFBP3*, and *CXCL8*) were enriched in KRAS signaling up, angiogenesis and VEGFA‐VEGFR2 signaling pathway, hypoxia, glycolysis, inflammatory response, toll‐like receptor signaling, and TGF‐beta receptor signaling (Supporting Information 6: Figure S3E)—hallmark pathways of mid‐stage cancer. The late‐stage upregulated genes (*TOP2A*, *GCLC*, *SPINK1*, and *CAPS*) were enriched in the NRF2 pathway (WP2884), NRF2‐ARE regulation, glutathione metabolism, gamma‐glutamyl cycle, NFE2L2 (NRF2) survival signaling induced by photodynamic therapy, xenobiotic metabolism, aryl hydrocarbon receptor netpath, and ferroptosis (Supporting Information 6: Figure S3F). These pathways directly mediate end‐stage cancer treatment resistance and survival, representing major contributors to therapy failure. Collectively, these findings demonstrate that the differentiation order and pivotal genes underlying LUAD are distinct from those of LUSC.

### 3.5. Epithelial‐Immune Cell Interactions and Communication Pathways in LUSC and LUAD

To assess the influence of distinct cell types on cellular crosstalk in LUSC and LUAD, separate “CellChat” analyses were performed. In LUSC, epithelial S3 cells exhibited the highest outgoing signaling activity, while macrophages exhibited the strongest incoming signaling among cell types. *SPP1*, *MIF*, *MK*, *ANNEXIN*, and *VISFATIN* were identified as key signaling ligands secreted by S3 cells, with macrophages serving as the target receptor cells (Figure 5A,B and Supporting Information 7: Table S4 and Supporting Information 8: Table S5). Enrichment analysis of these high‐confidence ligand–receptor pairs revealed significant involvement in pathways including coagulation, IL‐6/JAK/STAT3 signaling, inflammatory response, and the PI3K‐Akt signaling pathway WP4172 (Supporting Information 9: Figure S4A and Supporting Information 10: Table S6). In LUAD, epithelial S4 cells ranked highest in outgoing signaling patterns, while neutrophils showed the most robust incoming signaling activity. *MIF*, *GALECTIN*, and *ANNEXIN* emerged as core signaling ligands secreted by S4 cells, targeting neutrophils as receptor cells (Figure 5C,D and Supporting Information 11: Table S7 and Supporting Information 12: Table S8). Enrichment analysis of these pairs highlighted pathways such as interferon alpha response, allograft rejection, and interferon gamma response (Supporting Information 9: Figure S4B and Supporting Information 13: Table S9).

Figure 5Epithelial‐immune cell interactions and communication pathways in LUSC and LUAD. Heatmap illustrating the interaction strength between epithelial cells and immune cells in LUSC, with outgoing signaling patterns (A) and incoming signaling patterns (B) indicated. Heatmap showing the interaction strength between epithelial cells and immune cells in LUAD, with outgoing signaling patterns (C) and incoming signaling patterns (D) indicated. (E) Bar plot illustrating the pathway activity scores between epithelial cell subtype S3 and macrophages revealed that the MIF signaling pathway exhibited the highest activity among ligand–receptor pairs in LUSC. (F) Bar plot illustrating the pathway activity scores between epithelial cell subtype S4 and neutrophils revealed that the MIF signaling pathway exhibited the highest activity among ligand–receptor pairs in LUAD. Survival analysis comparing high and low expression groups of CD44 in LUSC (G) and CD74 in LUAD (H).

**Figure:**
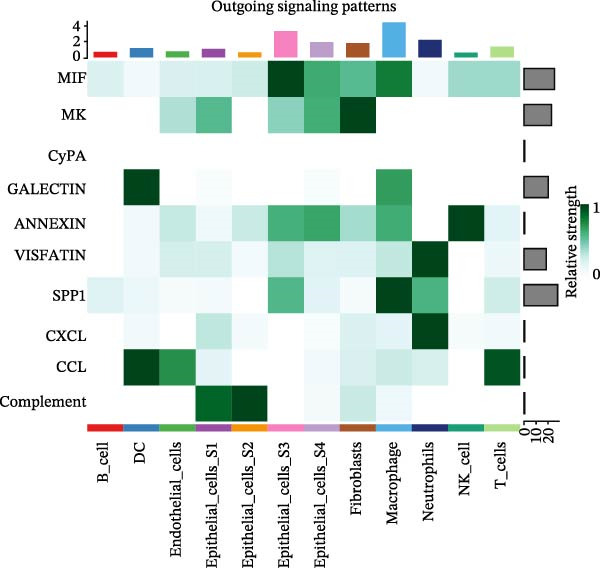
(A)

**Figure:**
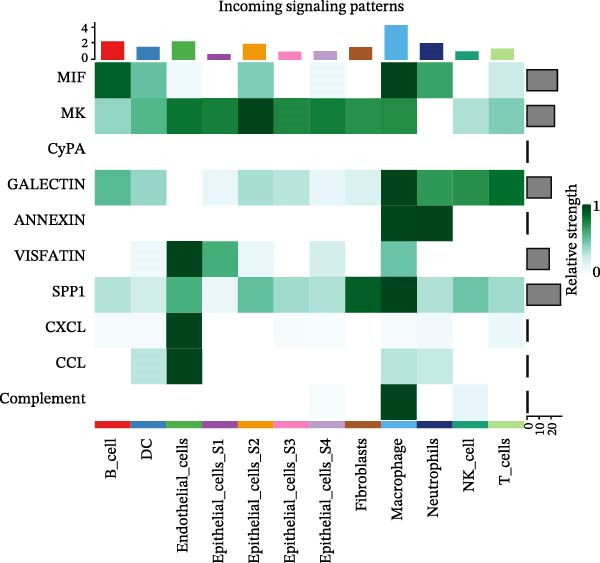
(B)

**Figure:**
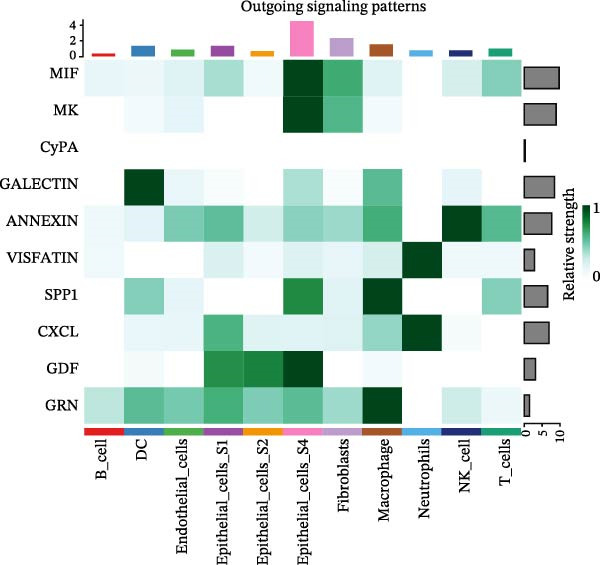
(C)

**Figure:**
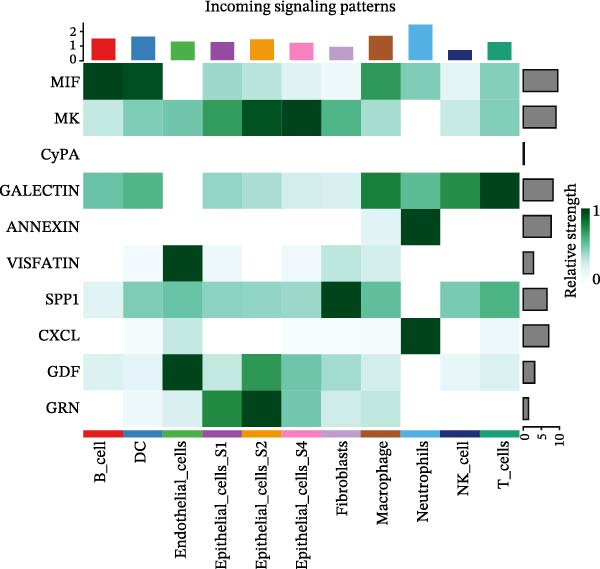
(D)

**Figure:**
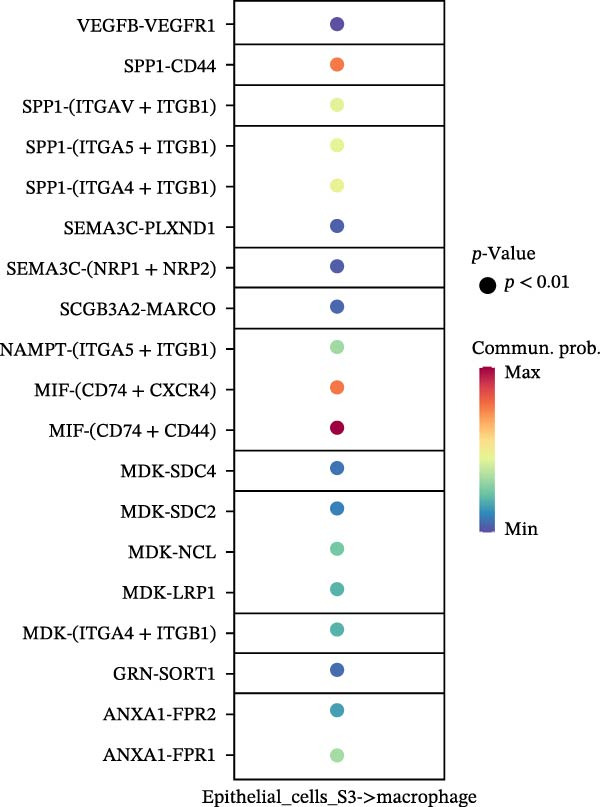
(E)

**Figure:**
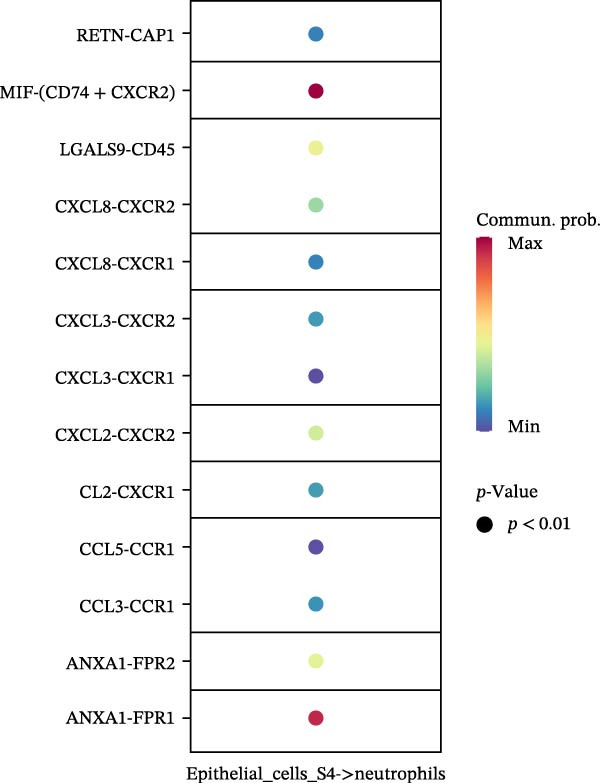
(F)

**Figure:**
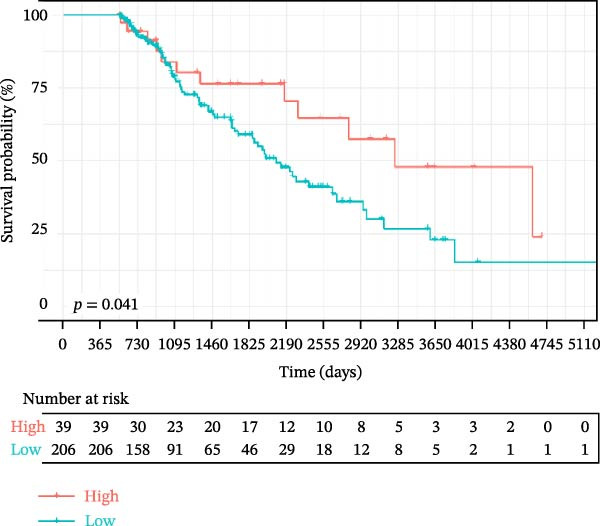
(G)

**Figure:**
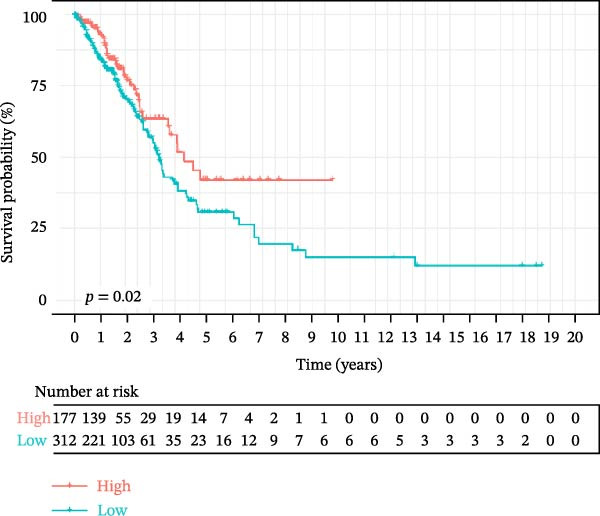
(H)

To quantify the communication strength of the highly scoring signaling pathways across tumor subtypes, we utilized the “computeCommunProbPathway” function to deduce signaling between S3 epithelial cells and macrophages in LUSC, as well as between S4 epithelial cells and neutrophils in LUAD. The MIF signaling pathway was found to be the most dynamically active in both LUSC and LUAD (Figure 5E,F and Supporting Information 14: Table S10). Enrichment analysis of ligand–receptor pairs within the MIF pathway showed significant enrichment in Interferon Alpha Response and Hypoxia in both LUSC and LUAD (Supporting Information 9: Figure S4C,D and Supporting Information 15: Table S11 and Supporting Information 16: Table S12). On this basis, we hypothesize that ligand–receptor genes within the *MIF* pathway may exert conserved functions across different tumor subtypes. To validate the “cellchat” results, we performed parallel analysis on epithelial subpopulations in the TME using the GSE117570 and GSE127465 datasets. The *MIF* pathway was confirmed as the most active signaling pathway in both LUSC and LUAD; in LUSC, S3 cells acted as the primary ligand‐source targeting macrophages (Supporting Information 17: Figure S5A,B); in LUAD, the strongest signaling crosstalk was observed between S4 ligand‐source cells and macrophage receptor cells (Supporting Information 17: Figure S5C,D). These results were consistent with our prior findings.

To explore the prognostic relevance of these ligand–receptor genes in LUSC and LUAD, we retrieved transcriptomic data from the TCGA database. Expression levels of *CD74*, *CXCR4*, *CXCR2*, *MIF*, and *CD44* differed significantly between normal and tumor samples (Supporting Information 9: Figure S4E,F). Survival analysis further demonstrated that *CD44* expression was significantly associated with LUSC prognosis, whereas *CD74* was significantly associated with LUAD prognosis (Figure 5G,H).

### 3.6. Macrophages are the Immune Cells That Exhibit the Highest Intercommunication Intensity in Both LUSC and LUAD

To elucidate which immune cells extensively crosstalk with epithelial cells in LUSC and LUAD, we analyzed intercellular communication within the TME. Immune cell composition profiling showed T cells as the most abundant subset in both tumors; additionally, macrophages and NK cells differed significantly in abundance between LUSC and LUAD, with neutrophils detected exclusively in LUAD (Supporting Information 18: Figure S6A). Using the “netAnalysis_signalingRole_scatter” function from the “cellchat” package to quantify immune cell communication intensity in the TME, we identified macrophages as the most interactive cell type (Figure 6A).

Figure 6Macrophages are the immune cells that exhibit the highest intercommunication intensity in both LUSC and LUAD. (A) Analysis of the communication intensity among immune cell types indicated that macrophages had the highest level of interactions in both LUSC and LUAD. (B,C) The pseudo‐temporal trajectory of macrophage subtypes in LUSC and LUAD is depicted, with the trajectory’s coloration corresponding to the pseudo‐time progression. (D) Distribution of the abundance of each macrophage cluster along the pseudo‐time trajectory. (E) Relative abundance of macrophage subtypes in LUSC (yellow) and LUAD (red) across the pseudo‐time trajectory. (F) Heatmap illustrating the pathway enrichment analysis of highly variable genes within each macrophage subtype. Pathway enrichment analysis of highly variable genes within each macrophage subtype (Macrophage 1‐G, Macrophage 2‐H, Macrophage 3‐I, and Macrophage 4‐J) in LUSC and LUAD.

**Figure:**
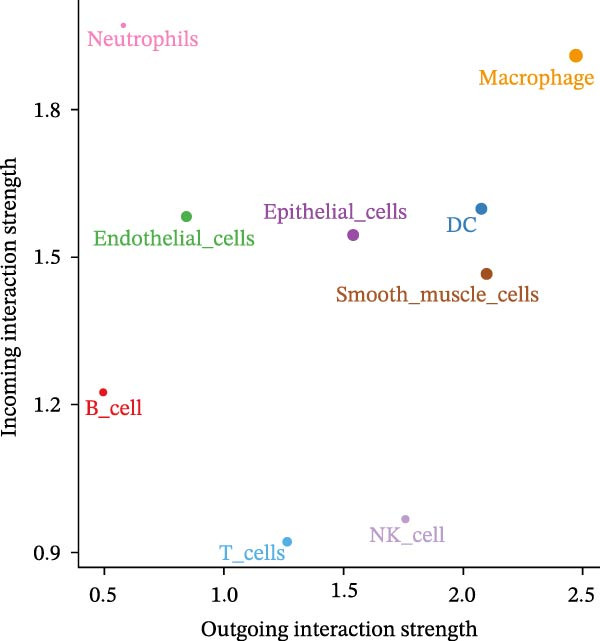
(A)

**Figure:**
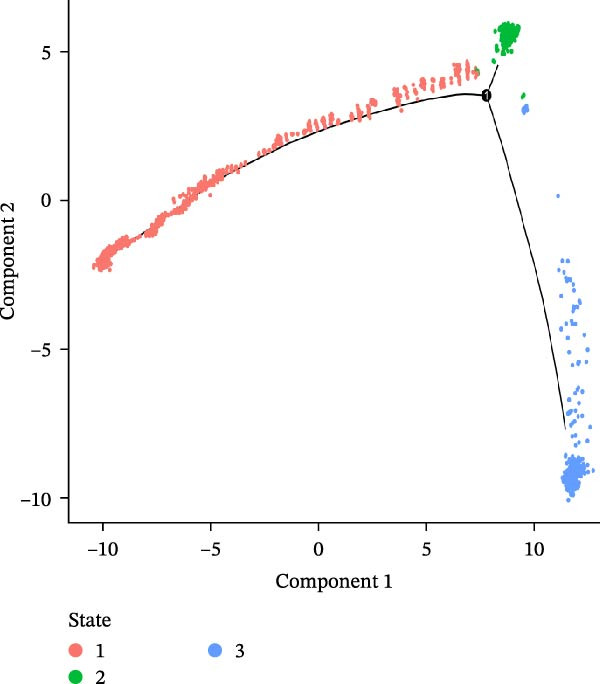
(B)

**Figure:**
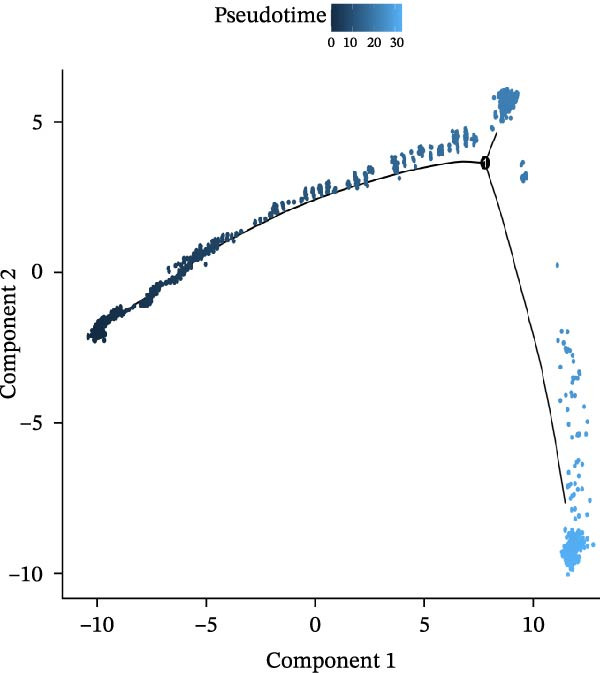
(C)

**Figure:**
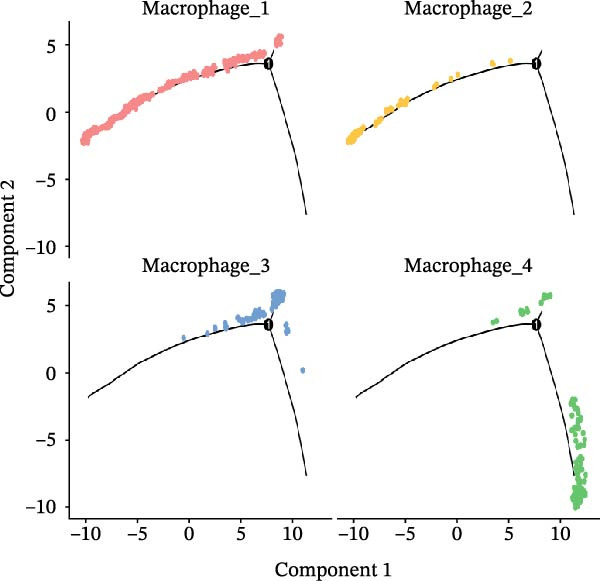
(D)

**Figure:**
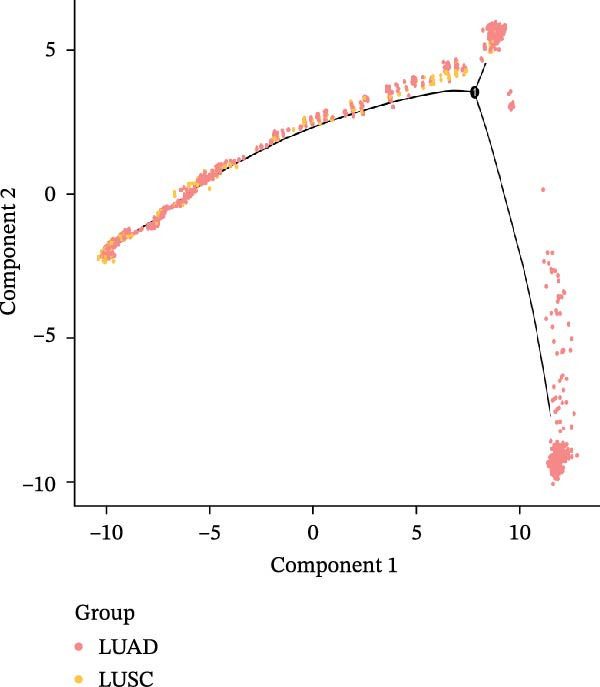
(E)

**Figure:**
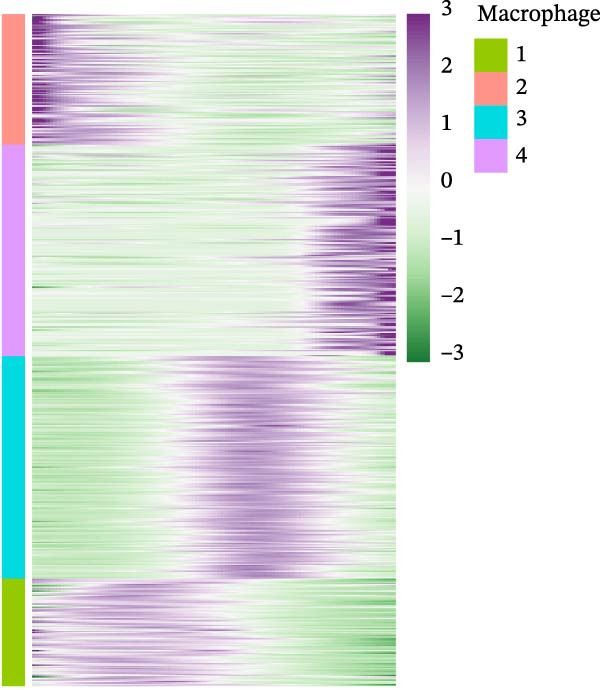
(F)

**Figure:**
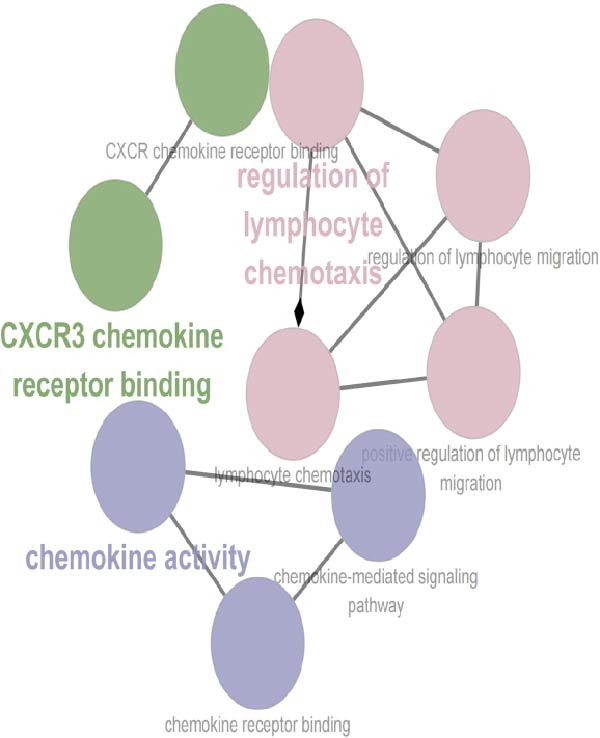
(G)

**Figure:**
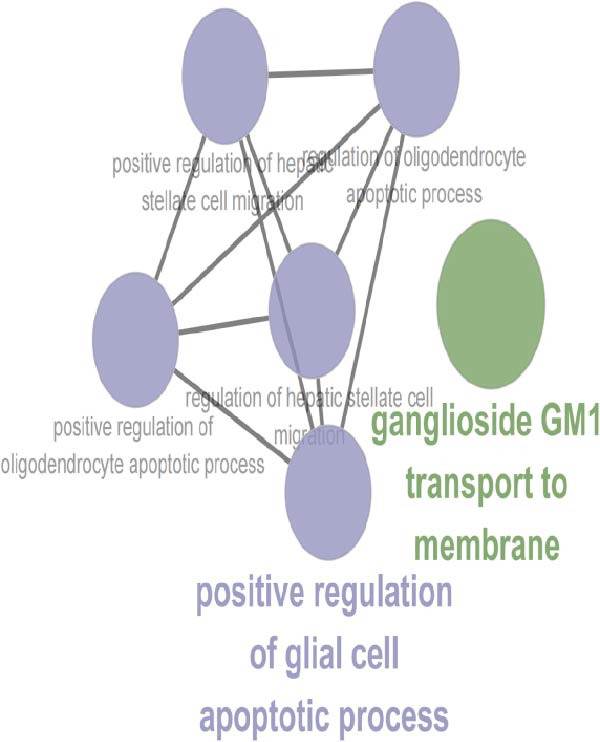
(H)

**Figure:**
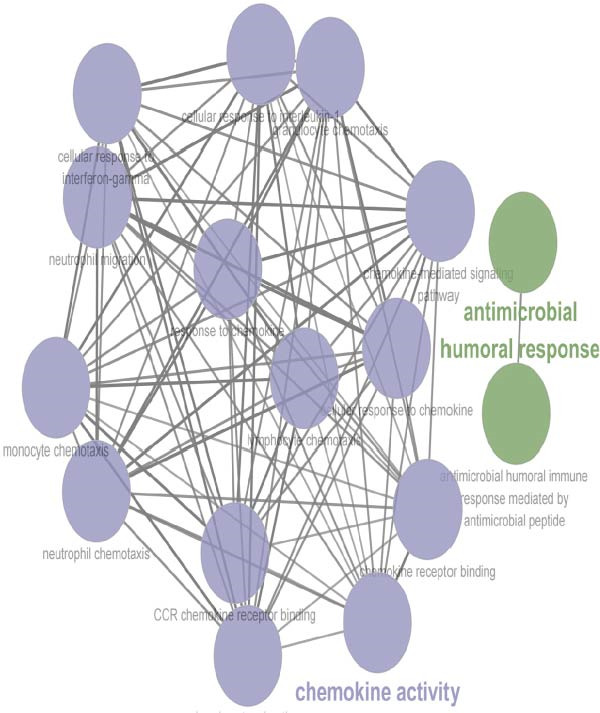
(I)

**Figure:**
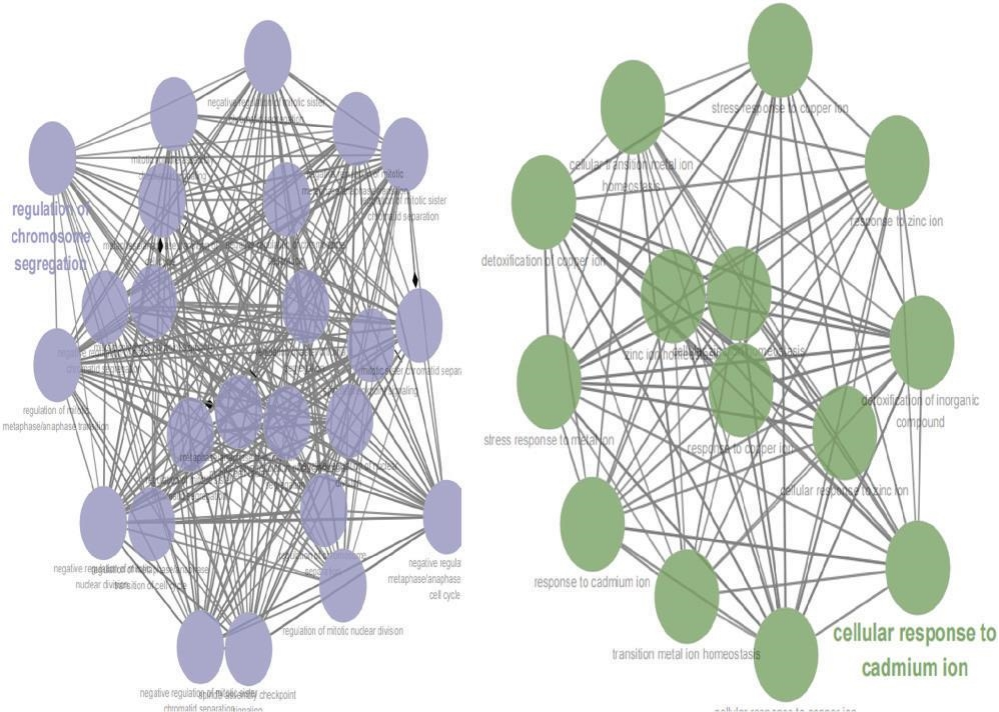
(J)

To characterize macrophage heterogeneity, we performed pseudotime analysis on macrophage subtypes. Macrophages were stratified into four subtypes following dimensionality reduction and batch effect correction using the Harmony algorithm (Supporting Information 18: Figure S6B). Monocle analysis categorized macrophage differentiation into three distinct stages (Figure 6B,C): Macrophage 1 and Macrophage 2 dominated the early stage (state 1), Macrophage 3 the middle stage (state 2), and Macrophage 4 the terminal stage (state 3) (Figure 6D). Notably, LUAD macrophages spanned all differentiation stages, whereas LUSC macrophages were restricted to the early stages (Figure 6E). The pseudotimeridge plot (Supporting Information 18: Figure S6C) and heatmap (Figure 6F) confirmed these differentiation patterns.

To investigate transcriptional and pathway dynamics across differentiation stages, ClueGO was utilized to perform enrichment analysis on HVGs of each macrophage cluster. Macrophage 1 was enriched in chemokine activity, *CXCR3* chemokine receptor binding, and lymphocyte chemotaxis regulation pathways (Figure 6G). Macrophage 2 showed enrichment in ganglioside GM1 membrane transport and positive regulation of glial cell apoptosis (Figure 6H). Macrophage 3 was associated with the antimicrobial humoral response and chemokine activity pathways (Figure 6I), while Macrophage 4 was enriched in cellular response to cadmium ions and chromosome segregation regulation (Figure 6J).

To identify stage‐specific upregulated marker genes, we performed pseudotime trajectory analysis of genes using the differential GeneTest function from the Monocle package. Macrophage 1 showed upregulated expression of CXCL10, CCL7, RND3, and FOLR3 (Supporting Information 18: Figure S6D), while Macrophage 2 showed upregulated IGHG3, CHI3L1, LTB, and AKAP12 (Supporting Information 18: Figure S6E). Macrophage 3 at state 2 was characterized by increased expression of CCL13, CCL18, CCL17, and FABP4 (Supporting Information 18: Figure S6F), and Macrophage 4 at state 3 displayed higher levels of CCNB1, MKI67, UBE2C, and MT1X (Supporting Information 18: Figure S6G). Furthermore, no terminal‐stage marker genes were upregulated in LUSC, a finding consistent with the restricted differentiation of LUSC macrophages—these cells were active only in early and mid‐differentiation stages and absent in the terminal stage. Pathway enrichment analysis revealed that Macrophage 1 markers (CXCL10, CCL7, RND3, and FOLR3) were enriched in pathways including IL‐6/JAK/STAT3 signaling, inflammatory response, TNF‐alpha signaling via NF‐κB, KRAS signaling up, Hippo‐YAP signaling pathway, and Hippo‐Merlin signaling dysregulation (Supporting Information 19: Figure S7A), which drive chronic inflammation, enhance cell survival, and promote early oncogenic transformation via mechanisms such as KRAS oncogene mutation and Hippo tumor suppressor pathway inactivation. Macrophage 2 markers (IGHG3, CHI3L1, LTB, and AKAP12) were enriched in IL‐6/JAK/STAT3 signaling, KRAS signaling up, and hypoxia (Supporting Information 19: Figure S7B); these pathways synergistically modulate inflammation, genetic mutation, and micro‐environmental remodeling to promote the formation of premalignant lesions.

Macrophage 3 markers (*CCL13*, *CCL18*, *CCL17*, and *FABP4*) were enriched in inflammatory response, fatty acid transporters, PPAR signaling pathway, and chemokine signaling pathway (Supporting Information 19: Figure S7C), mediating inflammation‐driven tumor progression, metabolic adaptation, and TME regulation during the transition from early to advanced tumor stages. Macrophage 4 markers (*CCNB1*, *MKI67*, *UBE2C*, and *MT1X*) were enriched in the G2‐M checkpoint, E2F targets, G1 to S cell cycle control, cell cycle, ATM signaling pathway, DNA damage response, and miRNA regulation of DNA damage response (Supporting Information 19: Figure S7D), which form a regulatory network that regulates the progression of advanced tumors.

### 3.7. Differential Roles and Signaling Pathways of Macrophage Subpopulations in LUSC and LUAD TME

To elucidate the functional roles of macrophages across tumor subtypes, the “CellChat” package was employed to analyze intercellular ligand–receptor interactions within LUSC and LUAD. The analysis indicated that Macrophages 1, 2, and 3 exhibited high bidirectional ligand–receptor communication intensity in both tumor types (Figure 7A,B and Supporting Information 20: Table S13 and Supporting Information 21: Table S14). In LUSC, Macrophage 4 exhibited negligible intercellular communication activity, with Macrophage 2 identified as the most interactive subtype; conversely, Macrophage 4 displayed the highest communication intensity in LUAD. These findings suggest that macrophage subpopulations may mediate distinct signaling pathways in the LUSC and LUAD TMEs, with potential implications for tumor initiation and progression. To further delineate the signaling patterns specific to macrophage subtypes, we applied the computeCommunProb function from the “CellChat” package to characterize intercellular interactions in LUSC and LUAD.

Figure 7Differential roles and signaling pathways of macrophage subpopulations in LUSC and LUAD TME. The interaction strength among different immune cell types and macrophage subtypes in LUSC (A) and LUAD (B). In LUSC, Macrophage 2 demonstrates the highest interaction intensity, while in LUAD, Macrophage 4 is the most interactive subtype. (C) Bubble chart showing the interaction strength between different macrophage subpopulations in LUSC. SPP1–CD44 ligand–receptor pair demonstrated the highest interaction intensity. (D) Network diagram showing the interaction between macrophage subpopulations and other cell types within the SPP1 signaling pathway in LUSC. (E) The functions of different cell types played within the SPP1 signaling pathway in LUSC. (F) Bubble chart showing the interaction strength between different macrophage subpopulations in LUAD. The RETN‐CAP1 ligand–receptor pair yielded the highest score for cellular interaction intensity. (G) Network diagram showing the interaction between macrophage subpopulations and other cell types within the RESISTIN signaling pathway in LUAD. (H) The functions of different cell types played within the SPP1 signaling pathway in LUAD.

**Figure:**
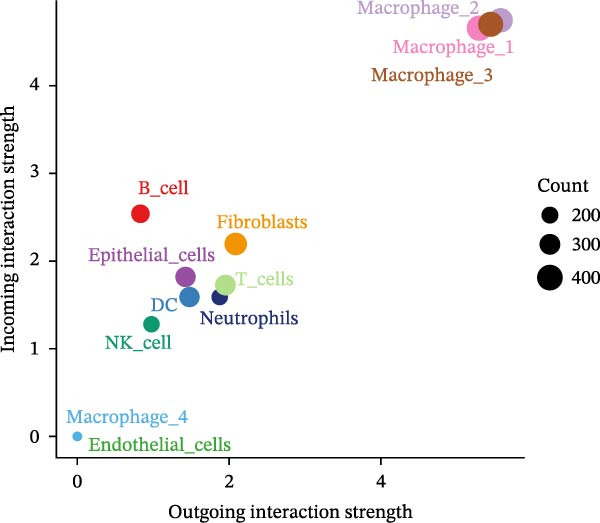
(A)

**Figure:**
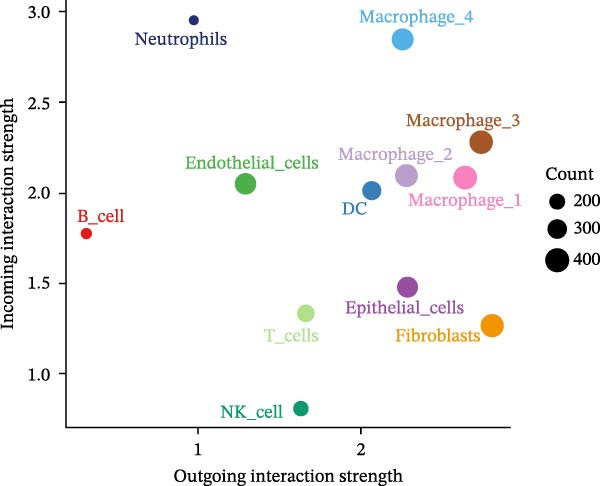
(B)

**Figure:**
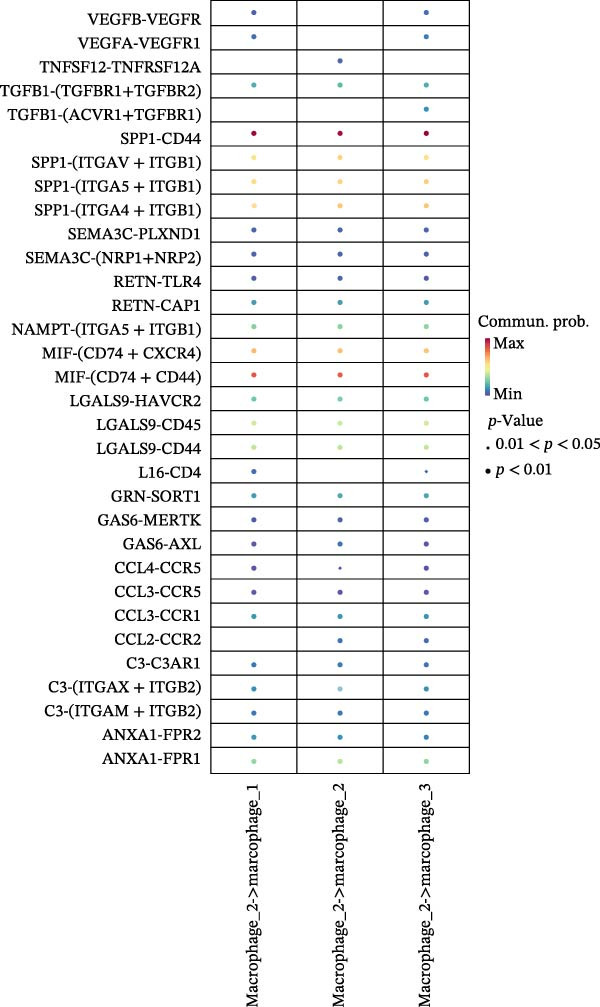
(C)

**Figure:**
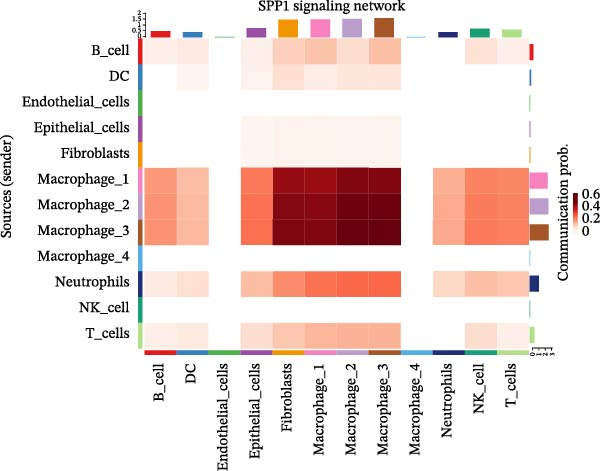
(D)

**Figure:**
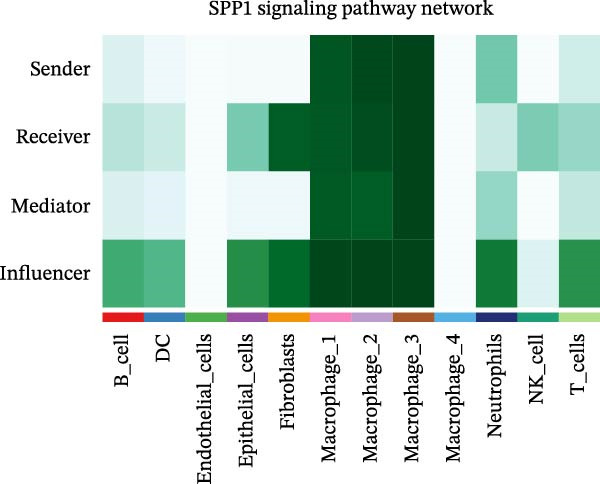
(E)

**Figure:**
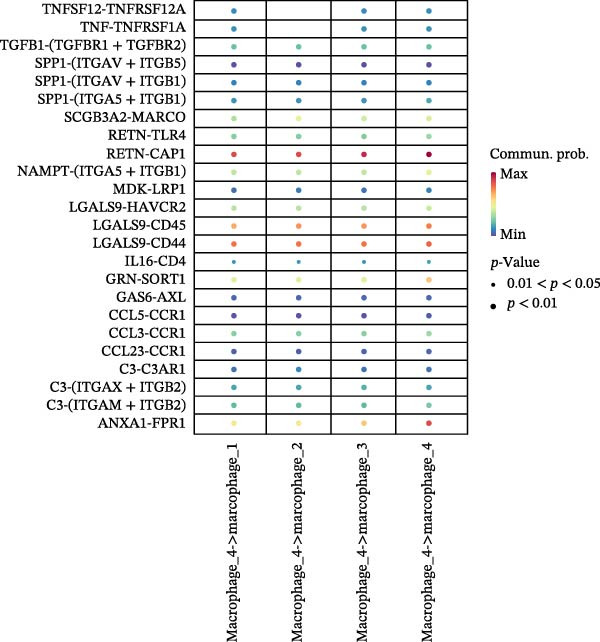
(F)

**Figure:**
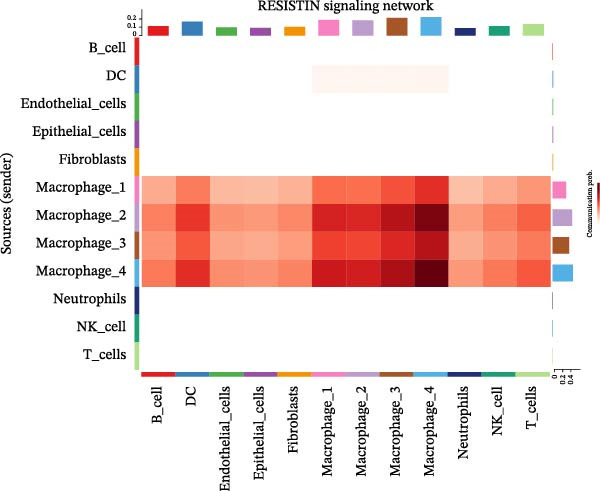
(G)

**Figure:**
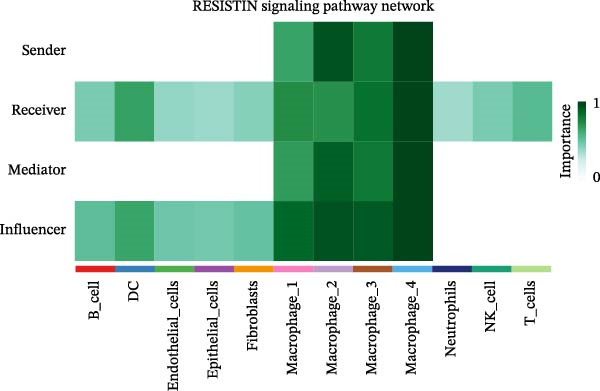
(H)

In LUSC, the *SPP1–CD44* ligand–receptor pair within the SPP1 signaling pathway exhibited the highest interaction intensity among macrophage subpopulations (Figure 7C). Subsequent analysis using the netVisual_aggregate function from the CellChat package identified Macrophages 1, 2, and 3 as the most interactive cell groups in this pathway. Unlike in LUAD, fibroblasts served as receptor cells with interaction levels second only to macrophages in LUSC SPP1 signaling (Figure 7D). Within the LUSC SPP1 pathway, macrophages acted as both signal senders and receivers, whereas fibroblasts functioned exclusively as receivers (Figure 7E). Notably, the *SPP1–CD44* interaction was the major contributor to SPP1 signaling activity. Consistent with this, our prior survival analysis of TCGA‐LUSC data demonstrated that *CD44* expression was significantly associated with LUSC prognosis, underscoring the functional relevance of the *SPP1–CD44* axis in LUSC.

To investigate macrophage contributions to the LUAD TME, the same analytical strategy was applied. The *RETN–CAP1* ligand–receptor pair in the RESISTIN signaling pathway exhibited the highest interaction intensity among LUAD macrophage subpopulations (Figure 7F and Supporting Information 22: Table S15). Macrophages 1–4 acted as ligand‐secreting cells, with other cell types serving as receptor cells in the RESISTIN signaling network (Figure 7G). Moreover, all four macrophage subtypes functioned as both senders and receivers in this pathway (Figure 7H), indicating the presence of autocrine regulatory activity—a finding consistent with previous reports [32]. This dual role enables macrophages to secrete cytokines for immune modulation and engage in paracrine crosstalk to regulate tumor immune pathways. To validate these CellChat findings, we reclassified macrophages in the GSE117570 and GSE127465 datasets and confirmed the presence of four macrophage subtypes via marker gene expression (Supporting Information 23: Figure S8A). The validation results corroborated the dominance of the SPP1 pathway (via *SPP1–CD44*) in LUSC, with Macrophage 2 signaling to Macrophages 1–3 (Supporting Information 23: Figure S8B,E). Similarly, a robust RESISTIN pathway (via *RETN–CAP1*) was observed in LUAD, with Macrophage 4 signaling to all other macrophage subtypes, which is consistent with our primary results (Supporting Information 23: Figure S8C,D).

## 4. Discussion

Although immunotherapy has moderately prolonged survival for patients with LUSC and LUAD to a certain extent, a substantial proportion of patients still exhibit primary or acquired resistance to such treatment [33, 34]. Thus, identifying novel and promising therapeutic targets for LUSC and LUAD has become a critical priority in current lung cancer research. In this study, we retrieved scRNA‐seq data of *NSCLC* from the GEO database, encompassing two samples of LUSC and nine samples of LUAD, for integrated analysis. We identified distinct epithelial and macrophage subpopulations with unique functional characteristics and defined their critical roles in tumorigenesis and progression. Furthermore, we identified MIF as a pivotal mediator orchestrating intercellular crosstalk between epithelial and immune cells.

Consistent with previous investigations [35], immune cells accounted for the predominant proportion of tumor‐infiltrating cells in our study. These results confirm immune cell infiltration in tumor tissues and imply their potential role in tumor initiation and progression within the TME. Our analysis identified four distinct epithelial cell subpopulations (S1–S4) in both LUSC and LUAD, with S3 being LUSC‐specific—a finding consistent with prior reports of tumor‐specific epithelial subsets across cancer types [31, 36]. InferCNV analysis further showed S3 exhibited the highest genetic variability among the epithelial clusters, suggesting that pathways enriched in this subset may drive LUSC tumorigenesis and progression; notably, the Reactive Oxygen Species (ROS) pathway was significantly enriched in S3. ROS, generated via both enzymatic and non‐enzymatic reactions, acts as a signaling molecule regulating transcription factors involved in cell differentiation, proliferation, autophagy, and apoptosis [37–39]. We thus hypothesize that S3‐related genes may alter ROS levels, inducing uncontrolled proliferation of epithelial cells and thereby promoting LUSC tumorigenesis.

Although LUSC and LUAD are distinct *NSCLC* subtypes [1], they may share conserved functional similarities. Cellular abundance quantification demonstrated that S1 and S2 were present in both tumors, with S2 being the dominant epithelial subset in both subtypes. Further analysis revealed marked activation of the nterferon gamma response pathway in both S1 and S2. IFN‐γ, a unique interferon family member primarily secreted by T lymphocytes and NK cells [40], plays a central role in innate and adaptive immunity against viral, bacterial, and tumor challenges. Notably, IFN‐γ signaling in the TME is crucial for antitumor immunity, as evidenced by the link between IFN‐γ pathway inactivation (via somatic mutation or homozygous deletion) and resistance to PD‐1/CTLA‐4 antibody therapies [41, 42]. Collectively, these findings highlight the interferon gamma response pathway as a potential key target for immunotherapy in both LUSC and LUAD [43].

Pseudotime trajectory analysis of epithelial cells uncovered distinct differentiation trajectories in LUSC and LUAD, implying that the progression of these two tumor subtypes is driven by divergent molecular mechanisms and characteristic genes. Furthermore, this analysis demonstrated that genes belonging to the CXCL chemokine family were involved throughout the entire epithelial cell differentiation process in both LUSC and LUAD. CellChat analysis of CXCL family genes in the TME showed that S3‐macrophage interactions were particularly active in LUSC, whereas S4‐neutrophil interactions dominated in LUAD, implying that CXCL family genes mediate crosstalk between epithelial cells and major tumor‐infiltrating phagocytes (macrophages and neutrophils). As core regulators of leukocyte migration, CXCL chemokines modulate tumor proliferation, metastasis, invasion, angiogenesis, and therapy resistance by orchestrating leukocyte recruitment in the TME [36]. Studies confirm strong associations between CXCL family genes and lung cancer; notably, CXCL1/2/3/5/8 promote lung tumorigenesis via regulating cell proliferation and angiogenesis [36]. ELR + CXC chemokines bind distinct receptors (CXCR1/2 vs. CXCR3/4/5/6/7), activating downstream pathways (e.g., MAPK, PI3K/Akt, STAT3, and NF‐ κB) to drive tumor progression [44]. Notably, CXCL9/CXCL10 may contribute to fatal COVID‐19 respiratory failure by recruitment of T cells and macrophages via a TNFα/IFN‐γ synergistic pathway in inflamed lungs [45]. Consistent with previous reports, our findings demonstrated that S3‐macrophage and S4‐neutrophil ligand–receptor pairs were markedly enriched in key pathways (PI3K‐Akt, TNF‐α/NF‐κB, and interferon gamma) in LUSC and LUAD, respectively. Collectively, these findings suggest that the S3 and S4 epithelial subpopulations may contribute to tumor development in LUSC and LUAD.

*MIF* signaling was found to be the pathway with the highest signaling intensity in cell communication. As a potent tumor‐promoting factor across major tumor subtypes (e.g., carcinoma, sarcoma), *MIF* exerts its oncogenic effects mainly through two mechanisms: first, binding to receptors like *CD74* to activate MAPK and PI3K/Akt pathways, which suppresses cancer cell apoptosis, upregulates cyclin expression, and accelerates cell proliferation [46–48]; second, upregulating matrix metalloproteinase (MMP) expression to enhance cancer cell invasion and metastasis [49]. Notably, gene knockout models have uncovered a functional paradox of MIF: its genetic deletion results in poorer tumor‐associated outcomes in certain contexts, implying inherent physiological protective roles. As a key mediator of innate immunity, MIF can activate antigen‐presenting cells (e.g., DCs) and drive Th1 immune responses in early tumorigenesis, which is critical for initiating effective antitumor T cell immunity [50]. This paradox is exemplified in urethane‐induced lung cancer models, where *MIF*‐knockout mice display higher tumor burdens, suggesting that complete *MIF* ablation disrupts immune homeostasis, impairs immune surveillance, and compromises the clearance of early cancerous cells [51]. Our finding that *MIF* is the core mediator of key epithelial‐immune crosstalk axes in LUSC and LUAD clarifies the subtype‐specific regulatory role of *MIF* in tumor progression, offering a promising subtype‐specific therapeutic strategy for disrupting tumor‐promoting intercellular communication.

Macrophages were identified as the most extensively interconnected immune cell subset in both LUSC and LUAD, indicating their central role in TME immune regulation. Notably, *CCL7*, *CCL13*, *CCL17*, and *CCL18* expression correlates strongly with macrophage differentiation status—previous studies have linked these genes to diverse pathological processes, such as *CCL7*‐mediated tumor promotion in metastatic renal cancer, *CCL13* association with severe childhood asthma, *CCL17*‐induced arthritis pain, and *CCL18*‐related immunosuppression and tumor immune evasion [52–55]. Our study showed that *CCL7* expression decreases with macrophage differentiation in LUSC but remains stable in LUAD; this subtype‐specific expression pattern suggests *CCL7* may serve as a candidate marker for LUSC and warrants further exploration as a potential immunotherapeutic target. In contrast, marked dynamic changes in *CCL13*, *CCL17*, and *CCL18* expression were observed in LUAD, likely reflecting its greater tumor heterogeneity and more complex TME compared to LUSC. These expression characteristics highlight *CCL13*, *CCL17*, and *CCL18* as promising candidates for further elucidating LUAD‐specific TME regulation and potential therapeutic interventions.

In LUSC, our analysis identified the SPP1 signaling pathway as the most active in intercellular interactions, with the *SPP1–CD44* ligand–receptor pair being the key contributor to this pathway activity. Consistent with this, our research demonstrated that *CD44* expression correlates closely with LUSC prognosis, while *CD74* expression is strongly associated with LUAD prognosis, suggesting that these two molecules may serve as subtype‐specific candidate biomarkers for LUSC and LUAD, respectively. Notably, *CD44*, a well‐recognized cancer stem cell marker, is frequently linked to tumor invasion, metastasis, and poor prognosis [56]. Similarly, *CD74* not only participates in antigen presentation but also exerts pro‐tumor effects via activating oncogenic signaling pathways through its cell surface expression [57]. Importantly, *CD74*‐targeted therapies (e.g., milatuzumab) have accumulated clinical experience in treating systemic lupus erythematosus [58], providing a feasible translational basis for exploring *CD74* as a potential target in lung cancer. To advance the clinical application of *CD44* and *CD74*, we propose that future studies should first validate the correlation between *CD44/CD74* expression and patient prognosis in multicenter cohorts using techniques such as multiplex immunofluorescence [59] and establish a standardized detection process comparable to that of estrogen receptor detection in breast cancer [60]. In summary, ultimately the goal is to apply *CD44/CD74* for tumor diagnosis and prognosis stratification and further develop novel combination targeted therapy strategies for patients with resistance to traditional chemotherapy or immunotherapy.

### 4.1. Limitations of the Study

This study only focused on LUSC and LUAD subtypes, with all analyses based solely on bioinformatics approaches. Notably, the lack of experimental validation (e.g., in vitro or in vivo experiments) to verify the key findings imposes certain limitations on the reliability and generalizability of the conclusions.

## Author Contributions

Xiaoyu Zhang and Yunlong Zhao designed the study, performed data analysis, and wrote the first draft of the manuscript. Yingying Wang, Xiaomin Yu, and Hongyu Xia participated in data collation. Meiru Li and Xiu‐An Yang designed the study, analyzed data, and wrote the manuscript.

## Funding

This work was supported by Initial Scientific Research Fund for High‐Level Talents of Chengde Medical University (Grant 201901).

## Disclosure

All content of the paper is an original work independently completed by the authors. All views, data, arguments, and conclusions have been carefully verified and confirmed by the authors, who assume full responsibility for the authenticity, accuracy, originality, and completeness of the manuscript. No individuals other than the listed authors contributed to the conception, design, execution, analysis, or interpretation of the research presented in this manuscript. No third‐party services (including but not limited to manuscript writing/editing, data analysis, figure illustration, or experimental technical support) that have not been formally acknowledged in the manuscript were engaged for the preparation of this work. All authors have read and approved the final version of the manuscript.

## Ethics Statement

The authors have nothing to report.

## Consent

The authors have nothing to report.

## Conflicts of Interest

The authors declare no conflicts of interest

## Supporting Information

Additional supporting information can be found online in the Supporting Information section.

## Supporting information


**Supporting Information 1** Table S1: Information of critical factors relevant to the patient.

**Supporting Information 2** Table S2: Marker gene for nine cell types.

**Supporting Information 3** Figure S1: Detailed analysis of epithelial cell signatures in LUSC and LUAD in GSE200972. (A) UMAP plot showing the clustering of 10,759 epithelial cells from LUSC and LUAD samples into 18 distinct groups. (B) The relative abundance of the four epithelial cell signatures in LUSC and LUAD samples. (C) Box plot showing the CNV variability of each signature determined by InferCNV analysis.

**Supporting Information 4** Table S3: Details of epithelial cells in LUSC and LUAD.

**Supporting Information 5** Figure S2: Detailed analysis of epithelial cell signatures in LUSC and LUAD in GSE117570 and GSE127465. (A) The UMAP plot shows the four subtypes of epithelial cells in the validation set. (B) The UMAP plot displays the expression pattern of *AKR1C2*. (C) The UMAP plot displays the expression pattern of CES1. (D) The relative abundance of the four epithelial cell signatures in LUSC and LUAD samples. (E) Heatmap showing the variability of different epithelial cell signatures as determined by InferCNV analysis, with T cells used as reference cells. (F) Box plot showing the CNV variability of each signature determined by InferCNV analysis.

**Supporting Information 6** Figure S3: Pathway enrichment of genes in pseudotime analysis of epithelial cell subtypes. (A) The pathway status enriched by early‐stage genes in LUSC. (B) The pathway status enriched by mid‐term stage genes in LUSC. (C) The pathway status enriched by last‐stage genes in LUSC. (D) The pathway status enriched by early‐stage genes in LUAD. (E) The pathway status is enriched by mid‐term stage genes in LUAD. (F) The pathway status is enriched by last‐stage genes in LUAD.

**Supporting Information 7** Table S4: Outgoing cell patterns calculated using single‐cell data by CellChat in LUSC.

**Supporting Information 8** Table S5: Incoming cell patterns calculated using single‐cell data by CellChat in LUSC.

**Supporting Information 9** Figure S4: Detailed analysis of cell–cell communication in LUSC and LUAD. Pathway enrichment analysis of the ligand–receptor pairs involved in cell–cell communication between epithelial cells and immune cells in LUSC (A) and LUAD (B). Pathway enrichment analysis of the ligand–receptor pairs involved in the MIF signaling pathway in LUSC (C) and LUAD (D). (E) Violin plots illustrating the expression levels of CD74, CXCR4, MIF, and CD44 in normal and tumor samples of LUSC from the TCGA database. (F) Violin plots showing the expression levels of CD74 and CXCR2 in normal and tumor samples of LUAD from the TCGA database.

**Supporting Information 10** Table S6: Pathway enrichment analysis of LUSC high‐scoring ligand–receptor pairs through the hallmark and WikiPathways databases.

**Supporting Information 11** Table S7: Outgoing cell patterns calculated using single‐cell data by CellChat in LUAD.

**Supporting Information 12** Table S8: Incoming cell patterns calculated using single‐cell data by CellChat in LUAD.

**Supporting Information 13** Table S9: Pathway enrichment analysis of LUAD high‐scoring ligand–receptor pairs through the hallmark and WikiPathways databases.

**Supporting Information 14** Table S10: LUSC communication patterns calculated using single‐cell data by CellChat.

**Supporting Information 15** Table S11: Enrichment Pathways of MIF signaling of LUSC through hallmark and WikiPathways databases.

**Supporting Information 16** Table S12: Enrichment Pathways of MIF signaling of LUAD through the hallmark and WikiPathways atabases.

**Supporting Information 17** Figure S5: Validation of CellChat analysis for epithelial cells using the GSE117570 and GSE127465 datasets. (A) Activity of cell types in LUSC. (B) Pathway enrichment heatmap with epithelial cluster S3 as ligand and macrophages as receptor in LUSC. (C) Activity of cell types in LUAD. (D) Pathway enrichment heatmap with epithelial cluster S4 as ligand and macrophages as receptor in LUAD.

**Supporting Information 18** Figure S6: Detailed analysis of macrophage subtypes in LUSC and LUAD. (A) The relative abundance of different immune cell types in LUSC and LUAD samples. (B) UMAP plot showing the clustering of macrophage cells from LUSC and LUAD samples into four distinct groups. (C) Ridge plot showing the expression abundance of macrophage subtypes in LUSC and LUAD across the pseudo‐time trajectory. (D) The expression of selected highly variable genes (CXCL10, CCL7, RND3, and FOLR3) with increased expression at Macrophage4 along the pseudo‐time trajectory. (E) The expression of selected highly variable genes (IGHG3, CHI3L1, LTB, and AKAP12) with increased expression at Macrophage 4 along the pseudo‐time trajectory. (F) The expression of selected highly variable genes (CCL13, CCL18, CCL17, and FABP4) with increased expression at Macrophage4 along the pseudo‐time trajectory. (G) The expression of selected highly variable genes (CCNB1, MKI67, UBE2C, and MT1X) with increased expression at Macrophage 4 along the pseudo‐time trajectory.

**Supporting Information 19** Figure S7: Pathway enrichment of genes in pseudotime analysis of macrophage subtypes. (A) The pathway status enriched by Macrophage 1 genes. (B) The pathway status enriched by Macrophage 2 genes. (C) The pathway status enriched by Macrophage 3 genes. (D) The pathway status enriched by Macrophage 4 genes.

**Supporting Information 20** Table S13: The role of macrophage clusters in LUSC.

**Supporting Information 21** Table S14: The role of macrophage clusters in LUAD.

**Supporting Information 22** Table S15: For cellular interaction intensity among the macrophage subpopulations.

**Supporting Information 23** Figure S8: Validation of CellChat analysis for macrophages using the GSE117570 and GSE127465 datasets. (A) The UMAP plot shows the four subtypes of macrophages in the validation set. (B) Pathway enrichment heatmap with Macrophage 2 as ligand and Macrophages 1, 2, and 3 as receptors in LUSC. (C) Pathway enrichment heatmap with Macrophage 4 as ligand and all macrophages as receptors in LUAD. (D) The strength of the RESISTIN signal. (E) The strength of the SPP1 signal.

## Data Availability

All datasets analyzed in this study are publicly available. The single‐cell RNA sequencing dataset used for lung cancer tissues was obtained from the GEO database (Accession Numbers GSE200972, GSE117570, and GSE127465). Bulk RNA‐seq datasets with overall survival information, including TCGA, GSE200972, GSE117570, and GSE127465, were retrieved from the GEO database (https://www.ncbi.nlm.nih.gov/geo/) and the TCGA data portal (https://portal.gdc.cancer.gov/). All data used in this study are available from the corresponding repositories, and no new datasets were generated. The processed data and scripts supporting the current findings are available from the corresponding author upon reasonable request. The details of the references are listed below. Song et al. [61] recently performed single‐cell transcriptome experiments investigating the molecular characteristics of NSCLC paired tissues (data accessible at the NCBI GEO database, Accession Number GSE117570). Wang et al. [5], recently performed single‐cell transcriptome experiments investigating the transcriptomic profiles of multiple primary lung cancers (data accessible at NCBI GEO database [35], Accession Number GSE200972). Zilionis et al. [62], recently performed single‐cell transcriptome experiments investigating the subsets of tumor‐infiltrating myeloid cells in non‐small cell lung cancer (data accessible at NCBI GEO database [62], Accession Number GSE127465). Heath, A.P., Ferretti, V., Agrawal, S. et al. The NCI Genomic Data Commons. Nat Genet 53, 257–262 (2021) https://doi.org/10.1038/s41588-021-00791-5 [63].IF: 29.0 Q1 B1
