# Supplementary figures and images for: Macrophage‐Mediated Cellular Communication Networks in Lung Squamous Cell Carcinoma and Adenocarcinoma Revealed by Single‐Cell Sequencing
Source: Mediators Inflamm. 2026 Feb 15;2026:9934067. doi: 10.1155/mi/9934067 (PMC12907464; doi:10.1155/mi/9934067)

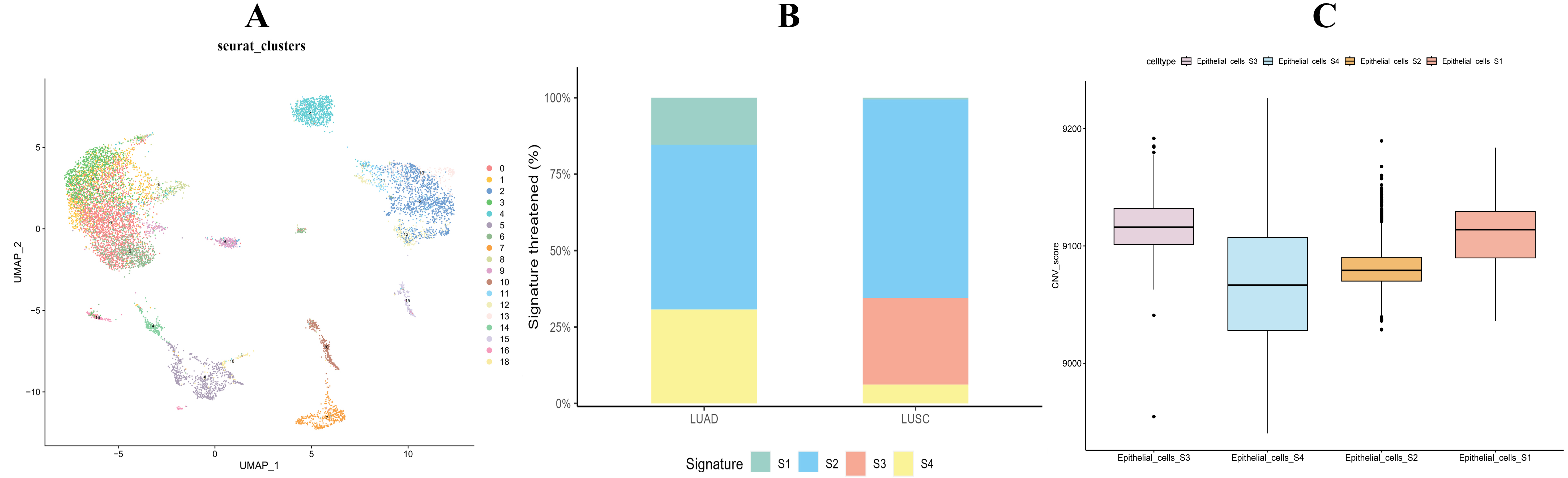

Supplement: Supplementary file 3 — Supporting Information 3 Figure S1: Detailed analysis of epithelial cell signatures in LUSC and LUAD in GSE200972. (A) UMAP plot showing the clustering of 10,759 epithelial cells from LUSC and LUAD samples into 18 distinct groups. (B) The relative abundance of the four epithelial cell signatures in LUSC and LUAD samples. (C) Box plot showing the CNV variability of each signature determined by InferCNV analysis. [file MI-2026-9934067-s003.jpg]

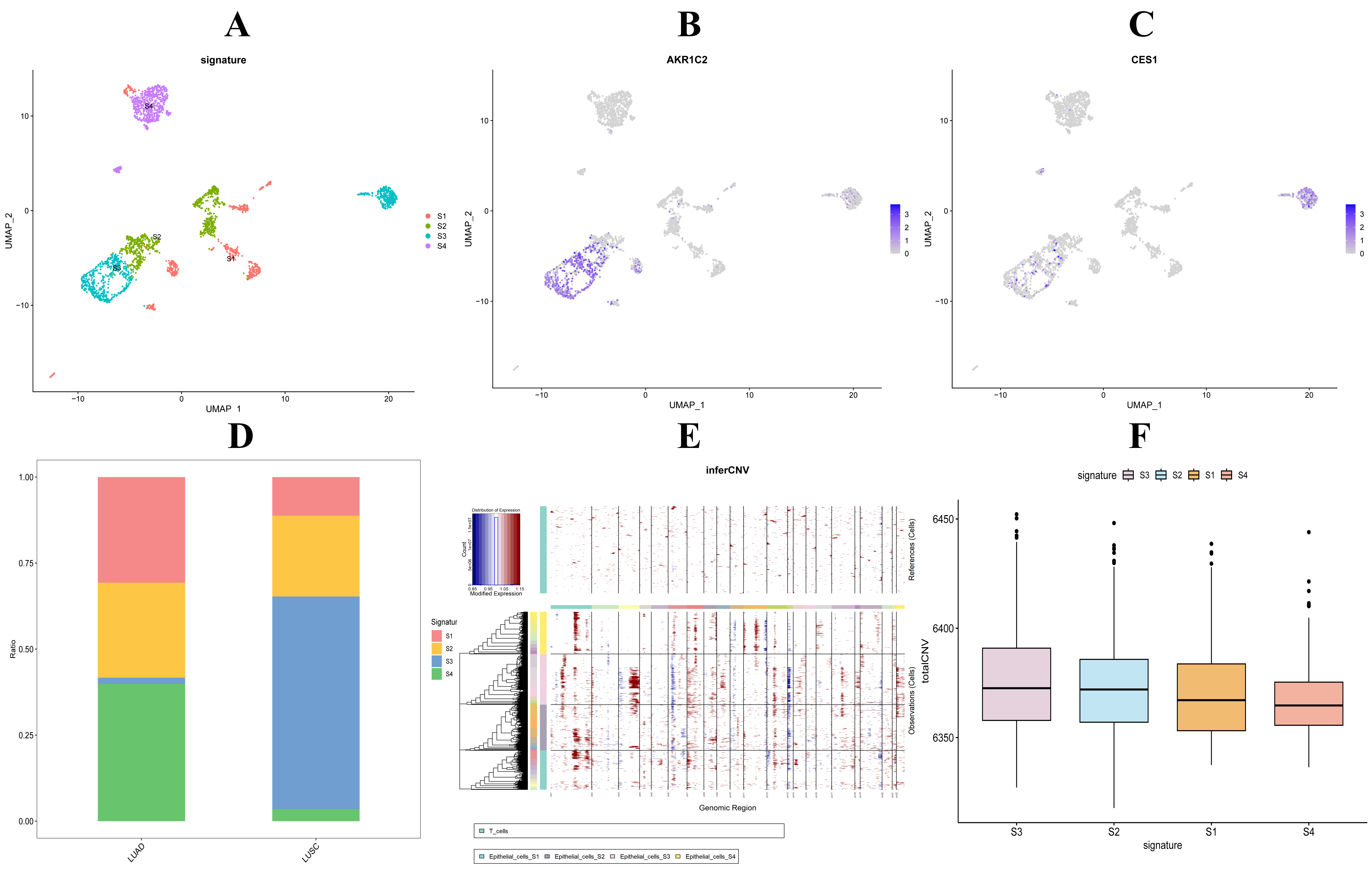

Supplement: Supplementary file 5 — Supporting Information 5 Figure S2: Detailed analysis of epithelial cell signatures in LUSC and LUAD in GSE117570 and GSE127465. (A) The UMAP plot shows the four subtypes of epithelial cells in the validation set. (B) The UMAP plot displays the expression pattern of AKR1C2. (C) The UMAP plot displays the expression pattern of CES1. (D) The relative abundance of the four epithelial cell signatures in LUSC and LUAD samples. (E) Heatmap showing the variability of different epithelial cell signatures as determined by InferCNV analysis, with T cells used as reference cells. (F) Box plot showing the CNV variability of each signature determined by InferCNV analysis. [file MI-2026-9934067-s005.jpg]

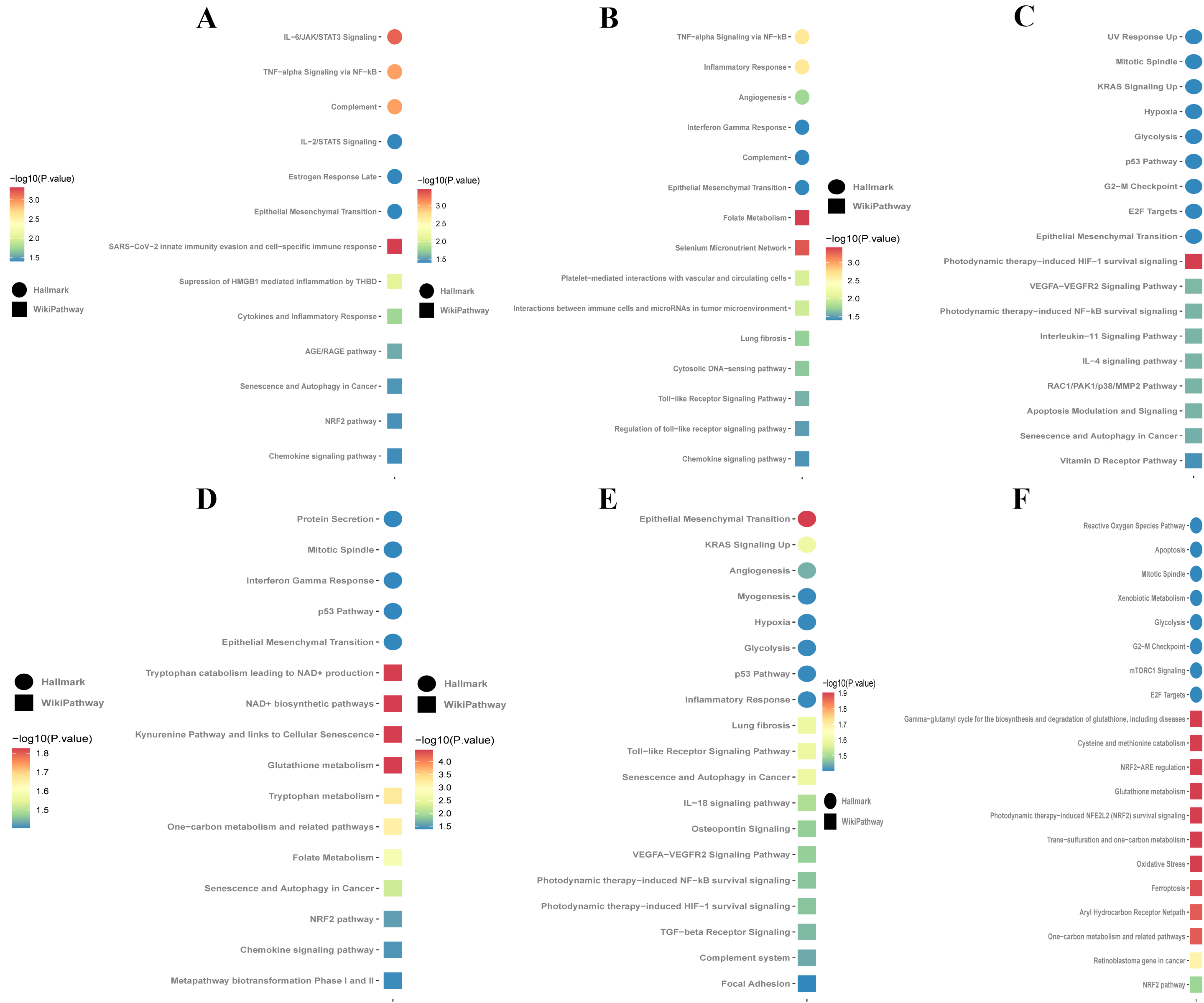

Supplement: Supplementary file 6 — Supporting Information 6 Figure S3: Pathway enrichment of genes in pseudotime analysis of epithelial cell subtypes. (A) The pathway status enriched by early‐stage genes in LUSC. (B) The pathway status enriched by mid‐term stage genes in LUSC. (C) The pathway status enriched by last‐stage genes in LUSC. (D) The pathway status enriched by early‐stage genes in LUAD. (E) The pathway status is enriched by mid‐term stage genes in LUAD. (F) The pathway status is enriched by last‐stage genes in LUAD. [file MI-2026-9934067-s006.jpg]

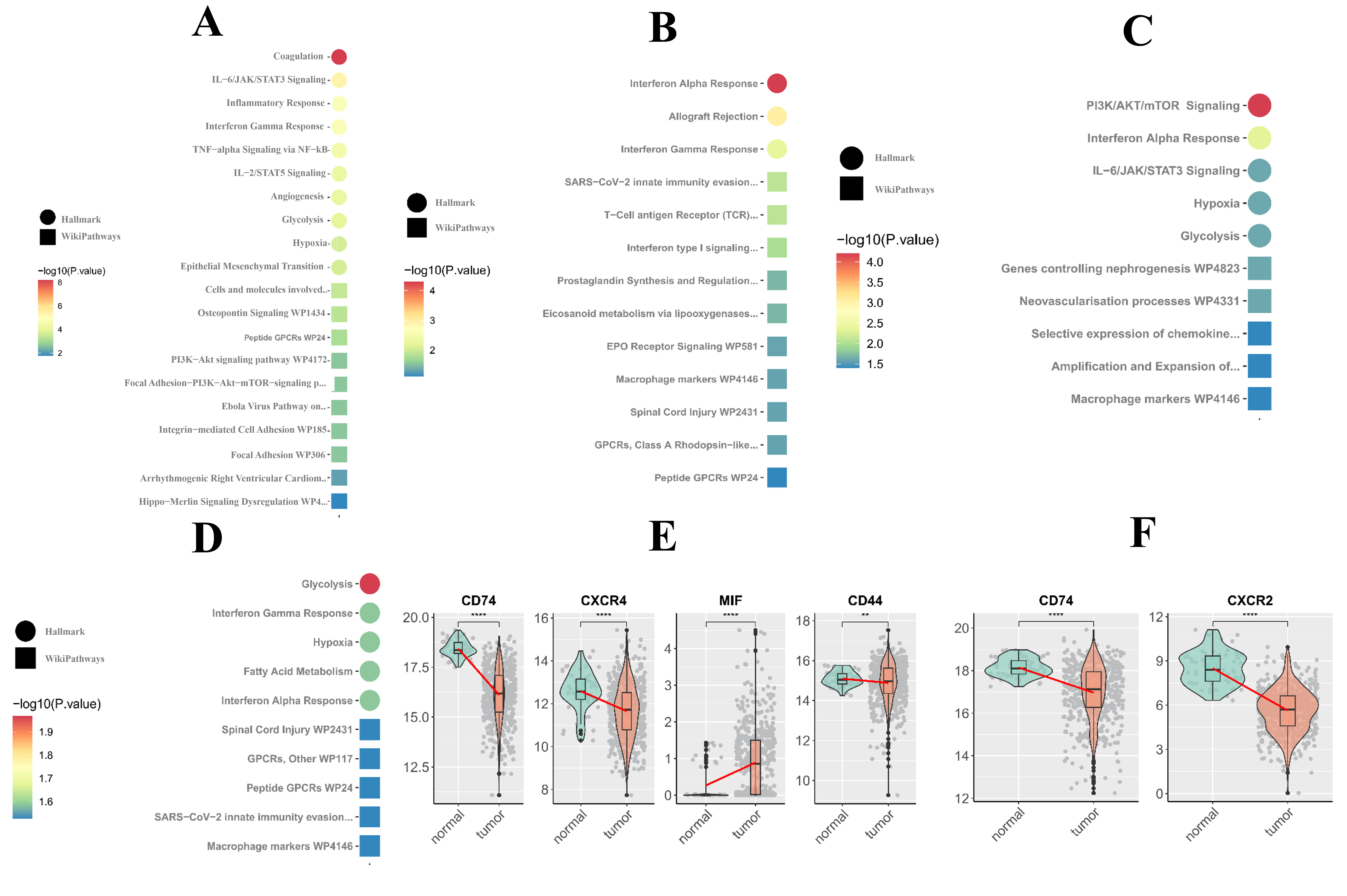

Supplement: Supplementary file 9 — Supporting Information 9 Figure S4: Detailed analysis of cell–cell communication in LUSC and LUAD. Pathway enrichment analysis of the ligand–receptor pairs involved in cell–cell communication between epithelial cells and immune cells in LUSC (A) and LUAD (B). Pathway enrichment analysis of the ligand–receptor pairs involved in the MIF signaling pathway in LUSC (C) and LUAD (D). (E) Violin plots illustrating the expression levels of CD74, CXCR4, MIF, and CD44 in normal and tumor samples of LUSC from the TCGA database. (F) Violin plots showing the expression levels of CD74 and CXCR2 in normal and tumor samples of LUAD from the TCGA database. [file MI-2026-9934067-s009.jpg]

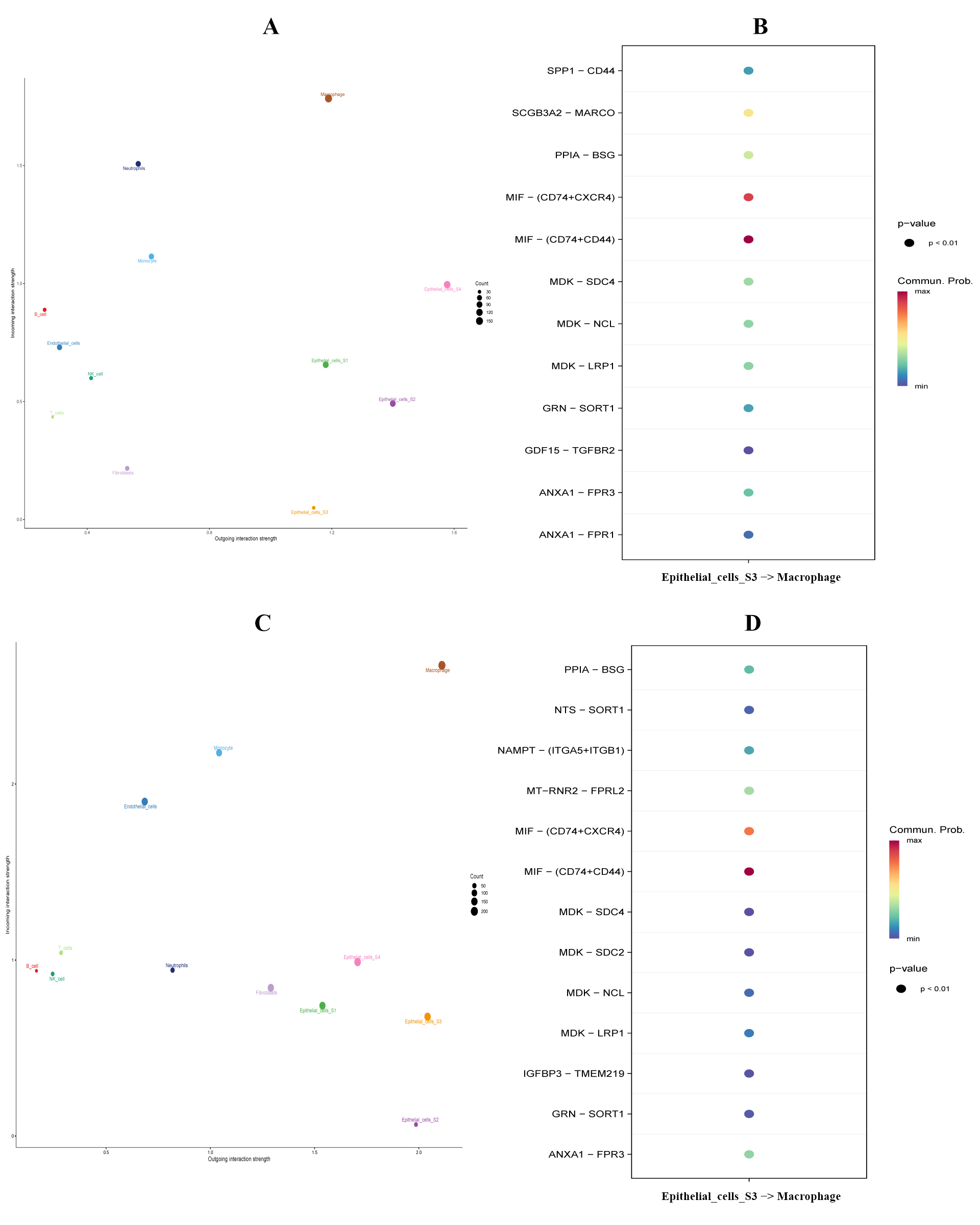

Supplement: Supplementary file 17 — Supporting Information 17 Figure S5: Validation of CellChat analysis for epithelial cells using the GSE117570 and GSE127465 datasets. (A) Activity of cell types in LUSC. (B) Pathway enrichment heatmap with epithelial cluster S3 as ligand and macrophages as receptor in LUSC. (C) Activity of cell types in LUAD. (D) Pathway enrichment heatmap with epithelial cluster S4 as ligand and macrophages as receptor in LUAD. [file MI-2026-9934067-s017.jpg]

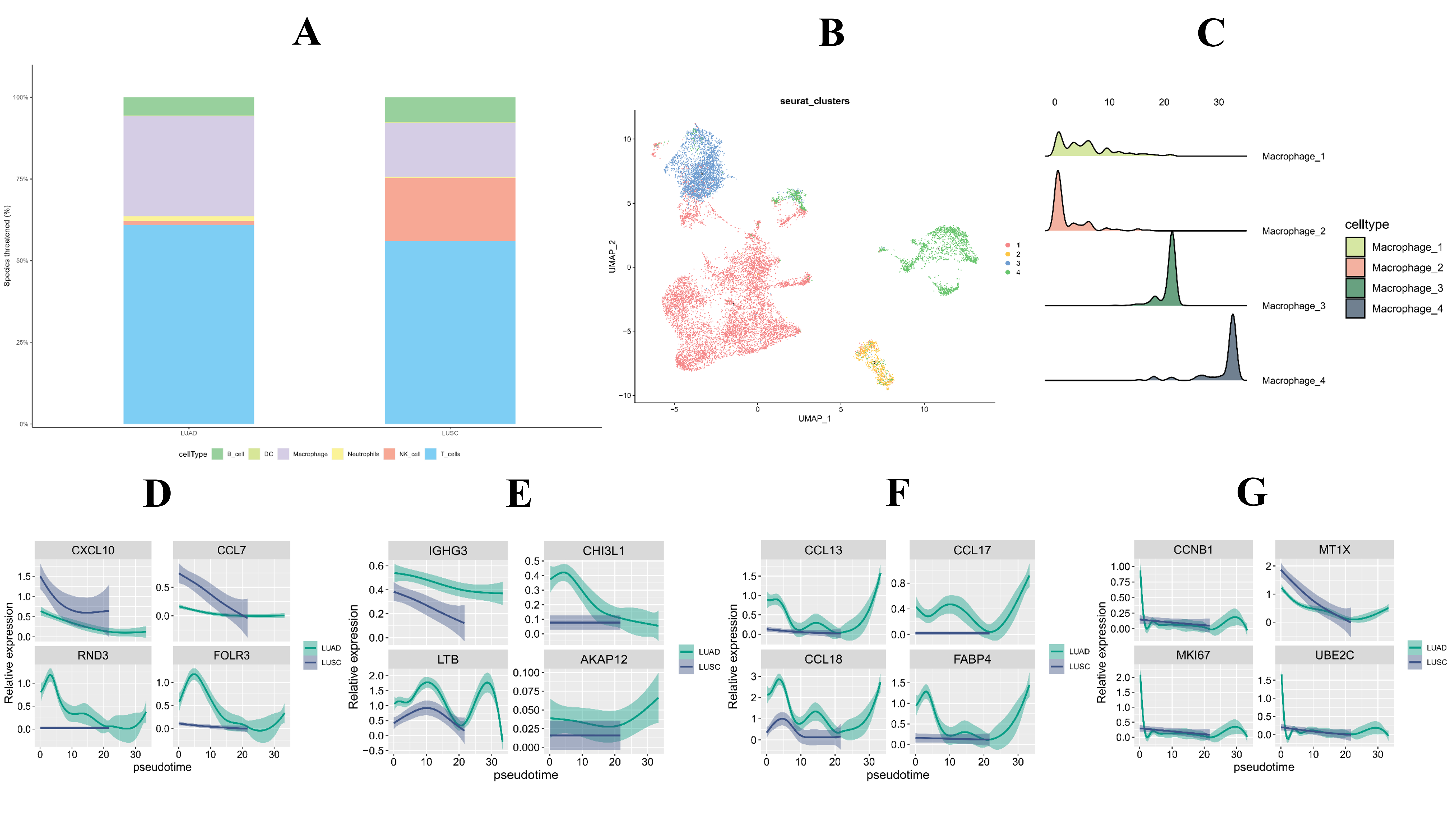

Supplement: Supplementary file 18 — Supporting Information 18 Figure S6: Detailed analysis of macrophage subtypes in LUSC and LUAD. (A) The relative abundance of different immune cell types in LUSC and LUAD samples. (B) UMAP plot showing the clustering of macrophage cells from LUSC and LUAD samples into four distinct groups. (C) Ridge plot showing the expression abundance of macrophage subtypes in LUSC and LUAD across the pseudo‐time trajectory. (D) The expression of selected highly variable genes (CXCL10, CCL7, RND3, and FOLR3) with increased expression at Macrophage4 along the pseudo‐time trajectory. (E) The expression of selected highly variable genes (IGHG3, CHI3L1, LTB, and AKAP12) with increased expression at Macrophage 4 along the pseudo‐time trajectory. (F) The expression of selected highly variable genes (CCL13, CCL18, CCL17, and FABP4) with increased expression at Macrophage4 along the pseudo‐time trajectory. (G) The expression of selected highly variable genes (CCNB1, MKI67, UBE2C, and MT1X) with increased expression at Macrophage 4 along the pseudo‐time trajectory. [file MI-2026-9934067-s018.tif]

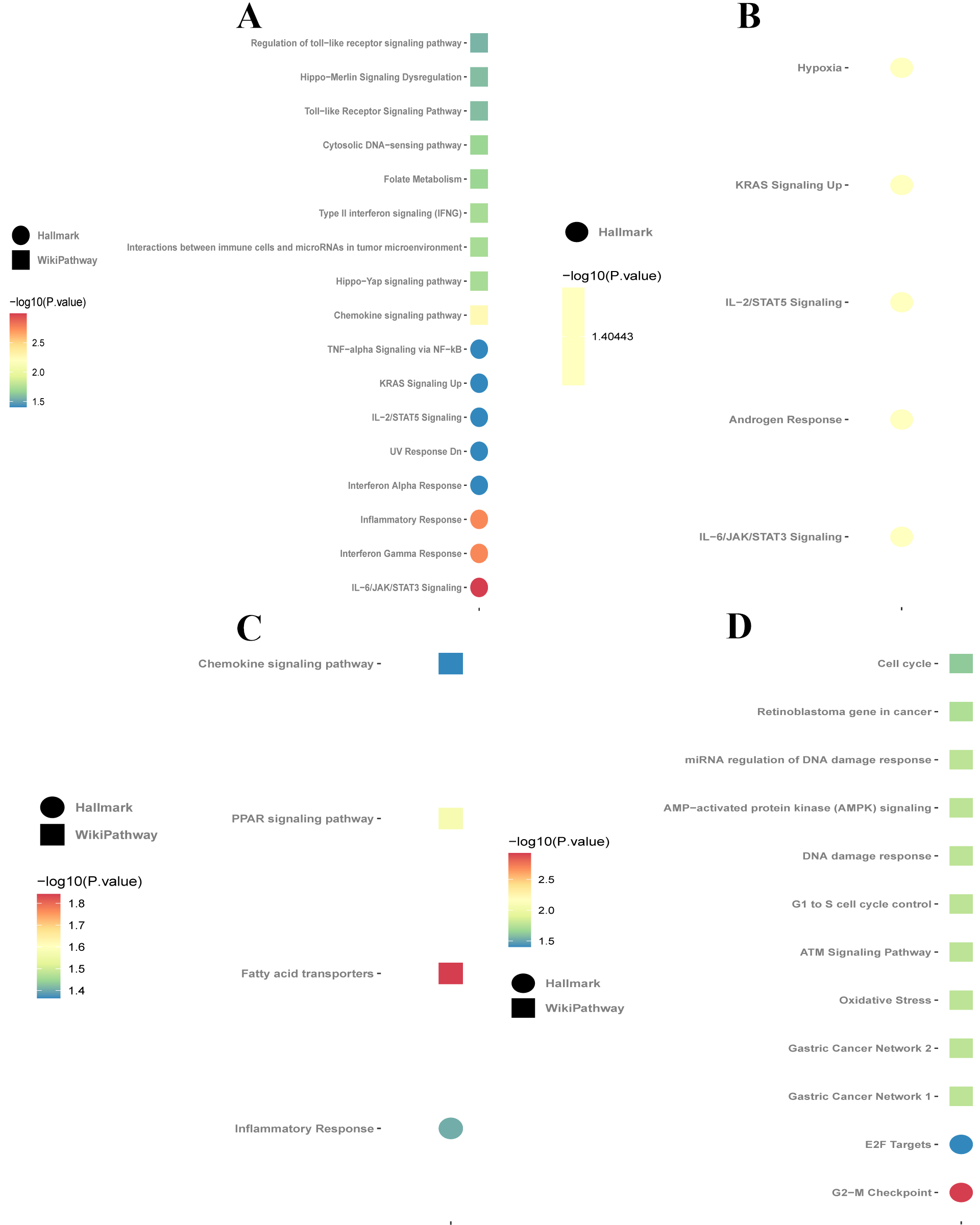

Supplement: Supplementary file 19 — Supporting Information 19 Figure S7: Pathway enrichment of genes in pseudotime analysis of macrophage subtypes. (A) The pathway status enriched by Macrophage 1 genes. (B) The pathway status enriched by Macrophage 2 genes. (C) The pathway status enriched by Macrophage 3 genes. (D) The pathway status enriched by Macrophage 4 genes. [file MI-2026-9934067-s019.jpg]

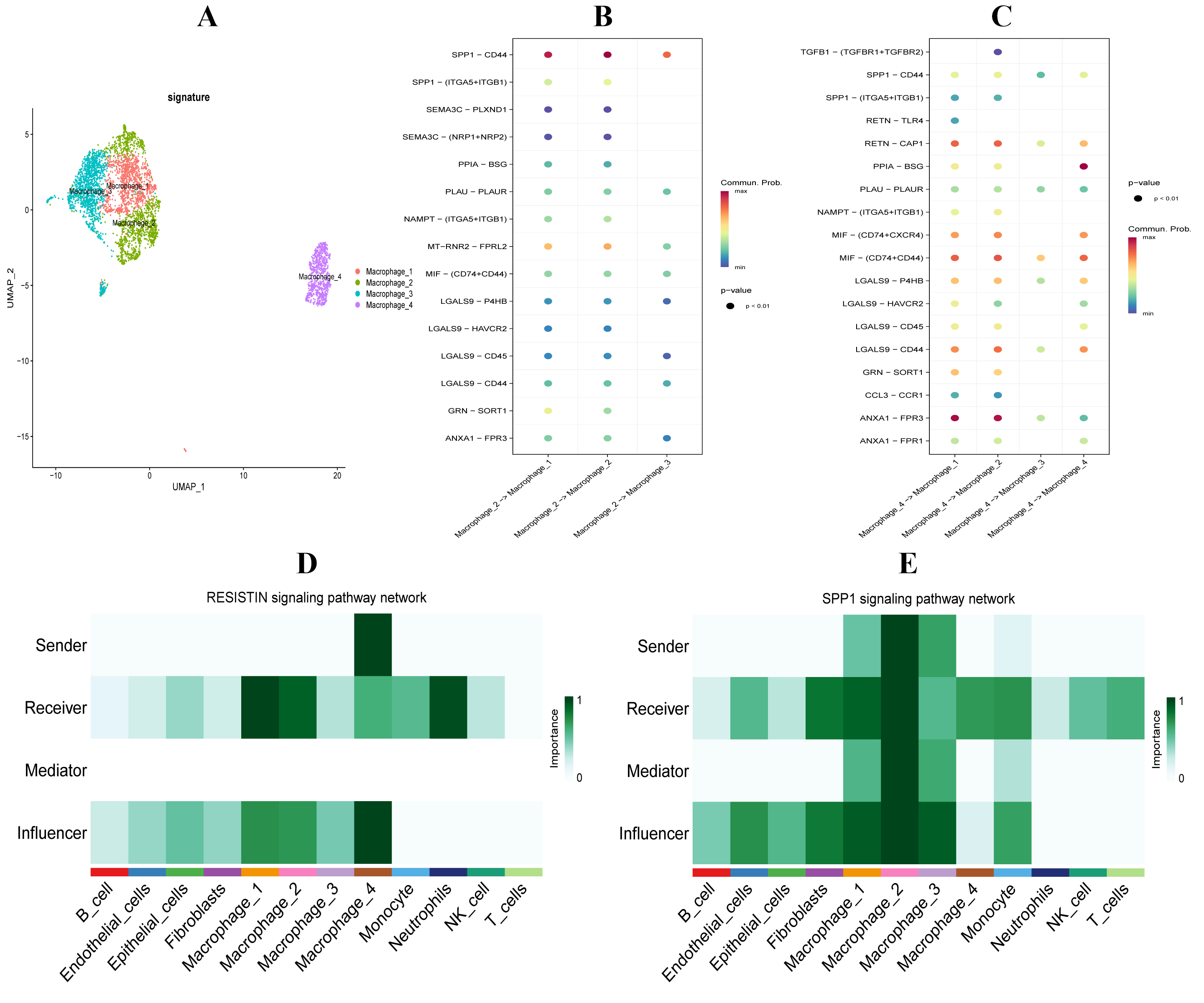

Supplement: Supplementary file 23 — Supporting Information 23 Figure S8: Validation of CellChat analysis for macrophages using the GSE117570 and GSE127465 datasets. (A) The UMAP plot shows the four subtypes of macrophages in the validation set. (B) Pathway enrichment heatmap with Macrophage 2 as ligand and Macrophages 1, 2, and 3 as receptors in LUSC. (C) Pathway enrichment heatmap with Macrophage 4 as ligand and all macrophages as receptors in LUAD. (D) The strength of the RESISTIN signal. (E) The strength of the SPP1 signal. [file MI-2026-9934067-s002.jpg]
